# Qualitative and Quantitative Analysis of the Major Constituents in WLJ Herbal Tea Using Multiple Chromatographic Techniques

**DOI:** 10.3390/molecules23102623

**Published:** 2018-10-12

**Authors:** Chao-Zhan Lin, Run-Jing Zhang, Yu-Feng Yao, Xiao-Dan Huang, Rong-Bo Zheng, Bo-Jian Wu, Chen-Chen Zhu

**Affiliations:** 1 Institute of Clinical Pharmacology, Guangzhou University of Chinese Medicine, No. 12 Jichang Road, Guangzhou 510405, China; linchaozhan@gzucm.edu.cn (C.-Z.L.); zhang_runjing@sina.com (R.-J.Z.); yao_yufeng@sina.com (Y.-F.Y.); 2 Research & Development Institute, Guangzhou Wanglaoji Pharmaceutical Company Limited, No. 831 Guanghuaer Road, Guangzhou 510450, China; zrbb0819@sina.com (R.-B.Z.); wu_bojian@sina.com (B.-J.W.)

**Keywords:** Wanglaoji herbal tea, UPLC-Q-TOF-MS, HPLC-MS/MS, evaporative light scattering detector

## Abstract

Quality control of Chinese herbal tea remains a challenge due to our poor knowledge of their complex chemical profile. This study aims to investigate the chemical composition of one of the best-selling and famous brand of beverage in China, Wanglaoji Herbal Tea (WLJHT), via a full component quantitative analysis. In this paper, a total of thirty-four representative constituents were identified or tentatively characterized using ultra-high performance liquid chromatography coupled with quadrupole tandem time-of-flight mass spectrometry (UPLC-Q-TOF-MS). Moreover, the quantitative analyses of fourteen constituents were performed by high performance liquid chromatography with a triple quadruple tandem mass spectrometry (HPLC-MS/MS) method and saccharide compositions of WLJHT were also quantitatively determined by high performance liquid chromatography (HPLC) with evaporative light scattering detector (ELSD) on a Hilic column, separately. Using multiple chromatographic techniques presented a good precision, sensitivity, repeatability and stability, and was successfully applied to analyze 16 batches of WLJHT samples. Therefore, it would be a reliable and useful approach for the quality control of WLJHT.

## 1. Introduction

Traditional Chinese herbal tea, developed by Chinese people during the long-term for disease prevention and health care under the guidance of traditional Chinese medicine, has been approved as an intangible cultural heritage by the Chinese government in 2006 [1,2,3].

Wanglaoji Herbal Tea (WLJHT), founded in the Daoguang eighth year of Qing Dynasty (Ad 1828), is the earliest Cantonese herbal tea and recognized as the ancestor of herbal tea. It is consisted of seven traditional Chinese medicines (*Chinese Mesona*, *PlumeriaeFlos*, *LoniceraeJaponicaeFlos*, *ChrysanthemiFlos*, *Prunellae Spica*, *Microctis Folium* and *Glycyrrhizae Radix et Rhizoma*), and widely used for heat-clearing, detoxicating, engender liquid and allay thirst. Modern pharmacological studies have proven its protective effects on liver damage [2], improvement of cytotoxic T Lymphocytes activity in spleen [4], amelioration on lipids metabolism [5] and glucometabolism [6], and enhancement of immune functions of restrain-stress mice [7]. In our previous phytochemical study [8], several polyphenol constituents as phenolic acids and flavonoids were separated and structural elucidated from WLJHT.

As “King of herbal tea”, WLJHT is lack of effective approach for quality control due to its chemical complexity. Three phenolic acids have been suggested to be employed for the quality assessment of WLJHT. However, as is known to all, multi-components in compositive herbs attribute comprehensive efficacy of herbal tea. Therefore, quality assessment based on a few of markers has been proven to be insufficient. In consideration of a mounts of unknown chemicals existed in herbal tea, LC/MS was widely used for the quality analyses of herbal medicines and herbal teas due to its powerful function on chemical structures [9]. Meanwhile, with the limitation of MS on carbohydrates, the evaporative light scattering detector (ELSD) has been used as an efficient method to identify and determine the little molecular carbohydrates in multi-herbals [10,11,12,13,14]. Therefore, complex application of multiple techniques for quality assessment of herbal tea seems to be more feasible and effective.

The aim of this study is to identify and quantify of the major constituents both the small molecules and saccharides in WLJHT, using ultra-high performance liquid chromatography (UPLC) coupled with quadrupole tandem time-of-flight mass spectrometry (Q-TOF-MS), and high performance liquid chromatography equipped with evaporative light scattering detector (HPLC-ELSD). 16 batches of samples were analyzed and the results were expected to provide comprehensive information for the quality control of WLJHT.

## 2. Results and Discussion

### 2.1. Identification of Constituents in WLJHT

**Figure 1:**
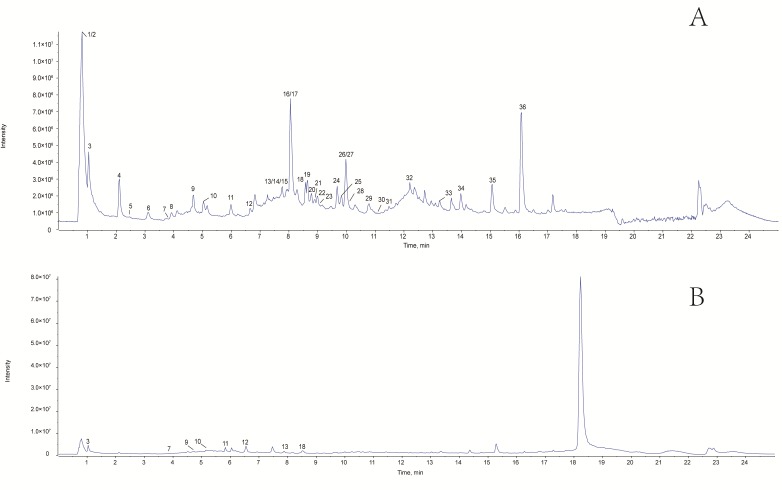
Representative total ion chromatograms of the WLJHT. (**A**) TIC of WLJHT sample in negative ion mode. (**B**) TIC of WLJHT sample in positive ion mode.

Both positive and negative ion modes were detected for MS analysis depending on the different chemical properties of WLJHT as shown in Figure 1A,B, respectively. In the negative-ion ESI mode experiments, the deprotonated molecules [M-H]^−^ were detected as the base peaks for most of the constituents. 

The exact molecular weight of each constituent was easily calculated according to the experimental mass of the pseudo-molecular ions, and the molecular formulas of those were deduced from each exact molecular weight obtained by Q-TOF-MS. The fragmentation information of each constituent was also obtained by Q-TOF-MS/MS as shown in Supplementary Information, which was quite useful for the identification of each constituent. Table 1 lists the retention time (t_R_), molecular formulas, experimental molecular weights, ESI-TOF-MS ions of thirty-four major peaks in the chromatograms came from WLJ herbal tea samples.

Among the thirty-six major constituents, a total of thirty-four constituents from the WLJHT were identified or tentatively characterized. They included 6 organic acids (Protocatechuid acid (**4**), Protocatechualdehyde (**5**), Chlorogenic acid (**9**), Caffeic acid (**10**), Isochlorogenic acid A (**23**), Rosmarinic acid (**27**)), 5 flavonoids (Rutin (**11**), Liquiritin (**16**), Keampferol-3-*O*-β-d-glucose-7-*O*-α-l-rhamnase (**19**), Narcissoside (**20**), Trifolin (**22**)), 2 triterpenoids (Macranthoidin B (**30**), Dipsacoside B (**34**)), and 1 iridoid (caffeoylplumieride (**18**)) was unambiguously identified by comparison of their t_R_s, ESI-IT-MS data with those of their reference substances. The other 20 compounds were tentatively characterized as follows: disaccharide (**1**), glucose acid (**2**), citric acid (**3**), 5-*O*-Caffeoylquinic acid (**6**), 15-demethylplumieride (**7**), syringic acid (**8**), isomeric di-O-CQA (**10**), cerberic acid B (**13**), violanthin (**14**), isoviolanthin (**15**), rosmarinic acid glycoside (**17**), isochlorogenic acid C (**21**), salvianolic acid B (**24**), isochlorogenic acid B (**26**), salvianolic acid A (**28**), salvianolic acid E (**31**), neriifolin (**32**), licorice saponin A3 (**33**), licorice saponin G2 (**35**), and glycyrrhizic acid (**36**) by comparing their exact molecular weights, MS^n^ spectra, UV absorptions and retention behaviors with those of reported compounds [8,9,15,16,17,18,19]. 

**Figure 2:**
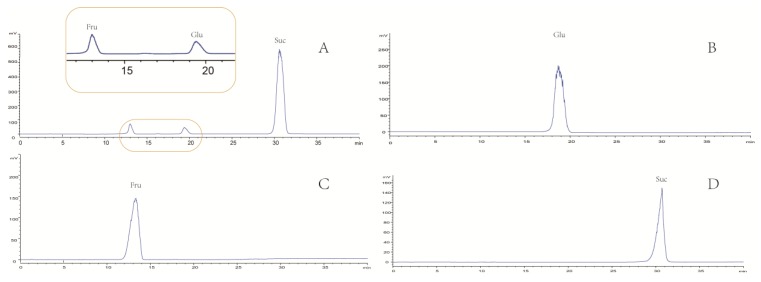
Comparative analysis of WLJHT (**A**) and 3 saccharides (**B**: Glucose; **C**: Fructose; **D**: Sucrose) using HPLC-ELSD couple with a Hilic column.

On the other hand, the saccharide profile was also shown in Figure 2, in which only four saccharides—fructose, a/β-d-glucose and sucrose—were identified by comparison with reference standards.

### 2.2. Limitation of Qualitative Analysis Solely Using Mass Spectrometry

WLJ herbal tea has two type components as non-sugar small molecules and little molecular carbohydrates. Although mass spectrometry is a powerful and sensitive analytical method as it can provide an accurate mass of the molecules and nanogram level of detection limit, solely using MS data in qualitative analysis is not suitable for weak electrolytes. For example, fructose, glucose and sucrose as the major carbohydrates of WLJHT, but they are too hard ionized to be detected by mass spectrometry. Therefore, it is suggested that the qualitative analysis is risky if only mass spectrometry is used. Moreover, the ionization mode plays an important role for MS analysis. The complex chemical composition of Chinese medicine requires a variety of ionization modes. Some components may be ignored if the single ionization mode is used. The comparison between two modes is necessary, which can avoid missing information.

### 2.3. Method Validation 

The linearity, ranges, regressions, LODs, LOQs and recoveries of the method are listed in Table 2. The data exhibited a satisfactory relationship between concentrations and peak areas of the analytes within the test ranges (*R*^2^ ≥ 0.9992). The RSDs of intra- and inter-day variations for 14analytes were not beyond3.19% and 4.75%, respectively. The LODs and LOQs ranged from 0.2 to 20 μg/L and from 0.5 to 30 μg/L, respectively. The established method demonstrated acceptable accuracy with spike recovery of 96.75%–105.78% for all analytes; and the RSDs of the peak areas for 14analytes detected within 24 h were lower than 4.87%. These results indicated that the developed UPLC-MS method was efficient, accurate and sensitive for simultaneous quantitative determination of the 14 constituents in WLJHT.

### 2.4. Quantitative Determination of the Major Constituents in the WLJHT

**Figure 3:**
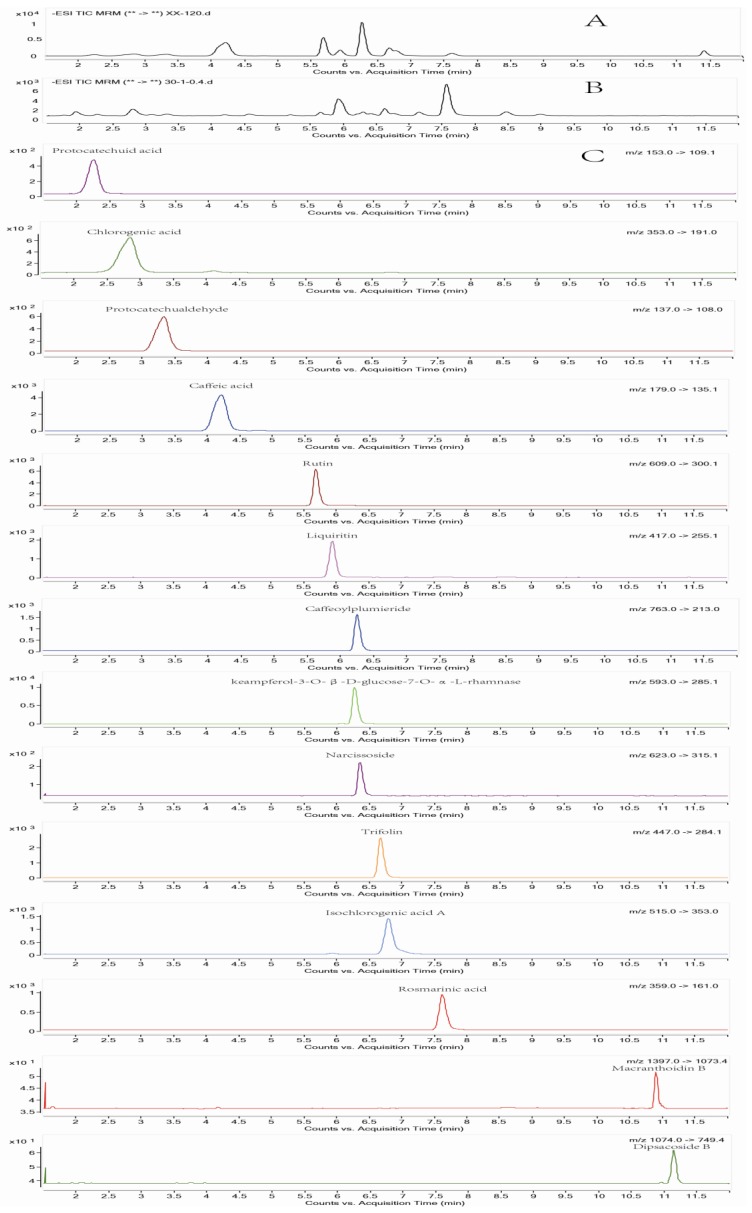
TIC chromatograms obtained by MRM for the negative-ion ESI triple quadrupole MS of 14 reference substance. (**A**) Extracted ion chromatograms of the fourteen reference substances; (**B**) Total ion chromatograms of the samples; (**C**) Extracted ion chromatograms obtained by MRM for negative-ion mode of the fourteen reference substances.

The above HPLC-ELSD and HPLC-MS/MS methods were applied to quantify the contents of the 17 major constituents in 16 batches of WLJHT samples (Figure 3). All of the contents were calculated by the external standard method, and the mean values and SDs from the three parallel determinations of each sample are summarized in Table 3. In general, the total content of known chemical components reached 78.61%–90.06% of the dry weight of WLJHT samples. Among them, 14 representative non-sugar small molecules possessed 0.25‰–0.29‰, and monosaccharide/sucrose accounted for 78.58%–90.03%. These results exhibited a general feature of WLJHT’s chemical profile: saccharides are the major components, and the non-sugar small molecules possess a very low content.

## 3. Materials and Methods

### 3.1. Materials and Reagents

Acetonitrile and methanol of HPLC grade were purchased from Honeywell (Muskegon, MI, USA). Water was purified by a Milli-Q water-purification system (Milford, MA, USA). Formic acid and ethyl acetate was analytical grade and purchased from Guangzhou Chemical Reagent Factory (Guangzhou, China).

**Figure 4:**
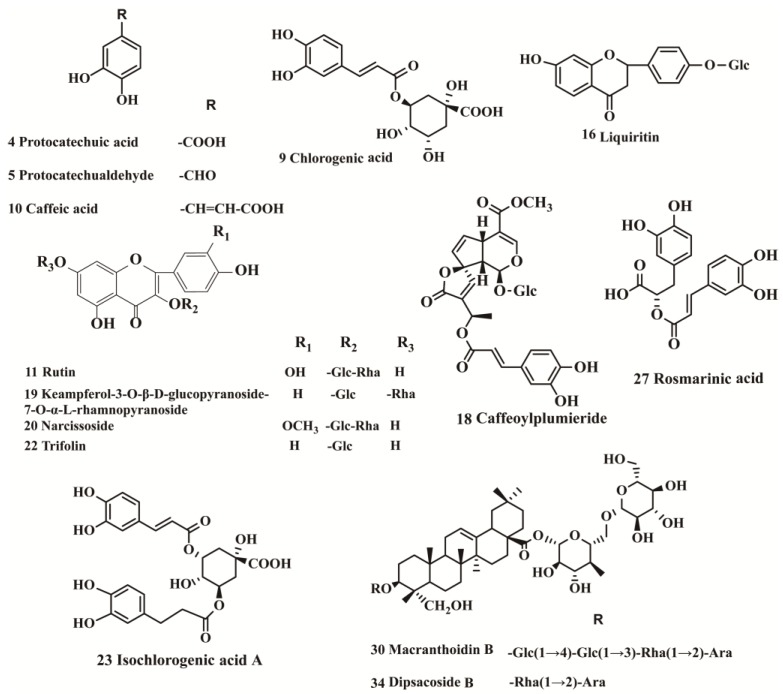
Chemical structures of the 14 reference substances.

Fourteen reference substamces, protocatechuid acid (**4**), protocatechualdehyde (**5**), chlorogenic acid (**9**), caffeic acid (**10**), narcissoside (**20**), trifolin (**22**), isochlorogenic acid A (**23**) and rosmarinic acid (**27**) were purchased from Chengdu Must Bio-Technology Co., Ltd. (Chengdu, China). Rutin (**11**), liquiritin (**16**), keampferol-3-*O*-β-d-glucose-7-*O*-α-l-rhamnase (**19**), macranthoidin B (**30**), and dipsacoside B (**34**) were purchased from Chengdu Push Bio-Technology Co., Ltd. (Sichuan, China). Caffeoylplumieride (**18**) were produced by our laboratory. Reference substances of d-(−)-fructose, d-(+)-glucose and sucrose were purchased from Sigma-Aldrich (St. Louis, MO, USA). Structures as shown in Figure 4 were elucidated based on their spectral analyses (IR, UV, MS and NMR), and their purities were found by HPLC analysis to be more than 98.0%.

Sixteen batches of WLJHT were provided by Guangzhou Wanglaoji Pharmaceutical Co., Ltd. (Guangzhou, China). The batch number for each sample was W141009, W141011, W141012, W141013, W141015, W141021, W141023, W141024, W141025, W141026, W141028, W141030, W141117, W141118, W141119, and W141120. Sample W141117 was used for our method development studies.

### 3.2. Preparation of Reference Solutions

Standard solutions of these 14 reference compounds were prepared in methanol-water (3:7, *v*/*v*) atthe known concentration (mg/mL):protocatechuid acid (2.01), protocatechualdehyde (1.46), chlorogenic acid (2.40), caffeic acid (2.02), Rutin (1.06), liquiritin (1.03), Caffeoylplumieride(1.36), keampferol-3-*O*-β-d-glucose-7-*O*-α-l-rhamnase (1.61), narcissoside (0.43), trifolin (0.42), isochlorogenic acid A (1.21), rosmarinic acid (1.98) macranthoidin B (0.21), and dipsacoside B (0.22). All standard solutions were stored at 4 °C until used, and finally filtered through a nylon membrane filter (0.22 μm, Phenomenex, Los Angeles, CA, USA) before analysis.

### 3.3. Preparation of Sample Solutions

The samples were stored in a refrigerator at 4 °C until used, and they warmed to room temperature, and filtered through a nylon membrane filter (0.22 μm, Phenomenex, USA) for qualitative analysis. For HPLC-MS/MS analysis, 1 mL of sample solution was transferred to 5-mL volumetric flask, brought up to volume with methanol-water (3:7, *v*/*v*) and filtered through a nylon membrane filter (0.22 μm, Phenomenex, USA) prior to use.

### 3.4. UPLC/Q-TOF-MS/MS Instrumentation and Methods

UPLC/Q-TOF-MS analysis was performed using an AB Sciex 5600 Triple-TOFTM mass spectrometer (AB Sciex, Redwood, CA, USA) coupled to a Shimadzu UPLC LC-30AD system (Kyoto, Japan) which were controlled with an Analyst^®^ TF 1.7 software (AB Sciex, Framingham, MA, USA). The chromatographic separations were accomplished on an ACQUITY UPLC BEH C18 column (Waters, Milford, MA, USA; 100 × 2.1 mm, 1.7 μm) with the column temperature kept at 25 °C. 0.1% formic acid (A) and acetonitrile (B) were served as the mobile phases at a flow rate of 0.35 mL/min under the following gradient elution mode: 0–2 min, 5% B; 2–3 min, 5%–10% B; 3–5 min, 10% B; 5–11 min, 10%–30% B; 11–20 min, 30%–60% B; 20–25 min, 60%–95% B. The injection volume was 5 μL, the column temperature was at room temperature. The mass spectrometer coupled with an electrospray ionization (ESI) sources was run in negative/positive ion and high sensitivity mode to acquire the TOF-MS. Meanwhile, accurate mass measurements were acquired with an automated calibration delivery system. After optimization, the nebulizer gas, heater gas and curtain gas were set at 55, 55 and 35 psi, respectively, and nitrogen was used as the source gases. The source temperature, ion spray voltage and declustering potential were set at 500 °C, ±4500 V and 100 V, respectively. For the IDA experiments, the collision energies were set at −45 eV and −25 eV, and the collision energy spread was set at 15 eV. TOF-MS spectra were obtained from 100 to 1500 Da followed by information dependent acquisition (IDA) scanning from 50 to 2000 Da. For further review of the mass spectrometric data for qualitative analysis, PeakView 2.0 Software (AB Sciex, Framingham, MA, USA) was used.

### 3.5. HPLC-ELSD Instrumentation and Methods

A previously reported HPLC-HILIC-ELSD method was used to determine the monosaccharides and oligosaccharides in TCM samples [2]. For quantitation of d-(−)-fructose, d-(+)-glucose and sucrose, an Agilent 1260liquidchromatography system (Agilent Technologies, Palo Alto, CA, USA), and Alltech3300 evaporative light scattering detector (Grace Alltech, Deerfield, IL, USA) coupled with a Merck ZIC-HILIC (4.6 mm × 200 mm, Merck, Tokyo, Germany) column at 30 °C were used. Water (A) and acetonitrile (B) were served as the mobile phases at a flow rate of 1.0 mL/min under the following gradient elution mode: 0–10 min, 85% B; 10–20 min, 85%–70% B; 20–40 min, 70%–55% B. The injection volume was 10 μL, the drift tube temperature of ELSD was set at 60 °C and the nitrogen flow rate of ELSD was set at 1.8 L/min. The gain number was equal to 1.

### 3.6. HPLC-MS/MS Instrumentation and Methods

For quantitation of 14 major constituents in the WLJHT, the HPLC-MS/MS analysis was performed using an Agilent 1260 HPLC system, equipped with G1312B 1260 Bin Pump, G1367E 1260 Hip ALS, G1316A 1260 TCC, Agilent 6460 LC/QQQ, Chemstation online workstation, electrospray ion source (ESI) and Agilent Poroshell 120 EC-C_18_ column (3.0 mm × 50 mm, 2.7 μm). 0.1% formic acid (A) and acetonitrile (B) were served as the mobile phases at a flow rate of 0.25 mL/min under the following gradient elution mode: 0–3 min, 13%–25% B; 3–7 min, 25% B; 7–8 min, 25%–40% B; 8–12 min, 40%–54% B. The injection volume was 5 μL, the column temperature was 25 °C. For MS condition, the capillary voltage was set at 3500 V, nozzle voltage was 500 V. Nebulizer air, drying-gas and sheath gas were all nitrogen, the drying-gas temperature was 300 °C and the flow rate was 5 L/min, the nebulizer pressure was 45 psi, sheath gas temperature was 300 and its flow rate was 11 L/min, the mass scanning range was set from *m*/*z* 100 to 1500. The optimum parameters of triple quadruple mass spectrometry are given in Table 2.

## 4. Conclusions

In this study, UPLC/Q-TOF-MS/MS, HPLC-MS/MS and HPLC-ELSD methods were developed for the identification and determination of the major constituents in WLJHT. The UPLC coupled with MS quickly identified or tentatively characterized 34 compounds in WLJHT based on their determined exact molecular weights and fragmentation patterns. Accurate determinations of 17 major constituents in WLJHT were performed by HPLC-ELSD and HPLC-MS/MS methods, respectively. Compared to the reported method in the literature [20], the complex application of the above three methods showed good stability, reproducibility, comprehensiveness, and could be applied for the quality control of WLJHT.

## Figures and Tables

**Table 1:** Identification of chemical constituents of WLJ herbal tea by UPLC/Q-TOF-MS/MS in positive and negative ion modes.

Peak	t_R_ (min)	Molecular Formula	Experimental Weight of Positive ESI-TOF-MS (*m*/*z*)/Error (ppm)/fragmental ion	Experimental Weight of Negative ESI-TOF-MS (*m*/*z*)/Error (ppm)/Fragmental Ion	Identification
1	0.80	C_12_H_22_O_11_	343.1238 [M + H]^+^/0.9	341.1089 [M − H]^−^/−0.1	Disaccharide
2	0.83	C_6_H_12_O_7_	343.1238 [M + H]^+^/0.9/240,183	341.1089 [M − H]^−^/−0.1/179, 161, 143, 113	Glucose acid
3	1.04	C_6_H_8_O_7_	Null ^c^	195.0514 [M − H]^−^/2.2/96, 87, 75	Citricacid ^b^, [15]
4	2.11	C_7_H_6_O_4_	Null	191.0202 [M − H]^−^/0.8/111, 87, 85, 67	Protocatechuid acid ^a^
5	2.45	C_7_H_6_O_3_	155.0338 [M + H]^+^/−0.5/85, 83, 56	153.0198 [M − H]^−^/3.0/109, 91, 81	Protocatechualdehyde ^a^
6	3.08	C_16_H_18_O_9_	139.0399 [M + H]^+^/7.0/95	137.0249 [M − H]^−^/3.8/93, 65	5-*O*-Caffeoylquinic acid ^b^, [9]
7	3.92	C_20_H_24_O_12_	Null	353.0872 [M − H]^−^/0.9/191, 179, 135	15-Demethylplumieride ^a^
8	3.95	C_9_H_10_O_5_	457.1333 [M + H]^+^ /−1.5/277	455.1188 [M − H]^−^/−1.4/275, 231	Syringic acid ^a^
9	4.68	C_16_H_18_O_9_	199.0597 [M + H]^+^/−1.8/139, 135, 107	197.0459 [M − H]^−^/2.2/135, 123	Chlorogenic acid ^a^
10	5.12	C_9_H_8_O_4_	355.1026 [M + H]^+^/0.8/163, 145	353.0874 [M − H]^−^/−0.8/191, 85	Caffeic acid ^a^
11	5.99	C_27_H_30_O_16_	181.0492 [M + H]^+^/−1.4/145, 135, 89	179.0351 [M − H]^−^/0.7/131, 85, 71	Rutin^a^
12	6.80	C_25_H_24_O_12_	611.1597 [M + H]^+^/−1.5/566, 548	609.1454 [M − H]^−^/−1.1/301	Isomeric di-*O*-CQA
13	7.91	C_10_H_10_O_5_	517.1324 [M + H]^+^/−3.0/499, 319, 163	515.1188 [M − H]^−^/−1.2/353, 191, 179	Cerberic acid B ^a^
14	7.91	C_27_H_30_O_14_	211.0597 [M + H]^+^/−1.5/193	209.0458 [M − H]^−^/1.4/165, 121, 119	Violanthin ^b^, [16]
15	7.92	C_27_H_30_O_14_	579.1708 [M + H]^+^ /−1.1/379, 337, 325	577.1556 [M-H]^−^/−0.3/457, 353	Isoviolanthin
16	8.05	C_21_H_22_O_9_	579.1701 [M + H]^+^/−1.1/507, 447, 337	577.1557 [M-H]^−^/−0.8/503, 473, 457, 413	Liquiritin ^a^
17	8.09	C_24_H_26_O_13_	419.1336 [M + H]^+^/−0.1/257, 137	417.1183 [M − H]^−^/−1.7/255, 148	Rosmarinic acid glycoside ^a^
18	8.58	C_30_H_32_O_15_	523.1437 [M + H]^+^/−1.6/181, 163	521.1294 [M − H]^−^/−1.0/359, 323	Caffeoylplumieride^a^
19	8.65	C_27_H_32_O_15_	633.1777 [M + H]^+^/−5.7/546, 474	631.1647 [M − H]^−^/−3.3/601/439, 163	Keampferol-3-*O*-β-d-glucose-7-*O*-α-l-rhamnase ^a^
20	8.78	C_28_H_32_O_16_	597.1776 [M + H]^+^/−6.2/548, 435	595.1659 [M − H]^−^/−1.5/285	Narcissoside ^a^
21	8.90	C_25_H_24_O_12_	625.1754 [M + H]^+^/−1.3/317	623.1612 [M − H]^−^/−0.7/315, 300	Isochlorogenic acid C
22	8.99	C_21_H_20_O_11_	517.1324 [M + H]^+^/−3.0/499, 319, 163	515.1188 [M − H]^−^/−1.2/353, 335, 173	Trifolin ^a^
23	9.00	C_25_H_24_O_12_	449.1077 [M + H]^+^/−0.2/287	447.0923 [M − H]^−^/−2.1/284, 255	Isochlorogenic acid A ^a^
24	9.68	C_36_H_30_O_16_	517.1324 [M + H]^+^/−3.0/499, 319, 163	515.1188 [M − H]^−^/−1.2/353, 191	Salvianolic acid B
25	9.83	C_48_H_68_O_5_	719.1604 [M + H]^+^/−0.3/643, 431	717.1458 [M − H]^−^/−0.4/673, 537, 519	Not identify
26	9.98	C_25_H_24_O_12_	725.5148 [M + H]^+^/1.2/661, 643	723.5029 [M − H]^−^/4.8/677	Isochlorogenic acid B
27	9.98	C_18_H_16_O_8_	517.1324 [M + H]^+^/−3.0/499, 319, 163	515.1188 [M − H]^−^/−1.2/353, 255, 203, 173	Rosmarinic acid ^a^
28	10.09	C_26_H_22_O_10_	361.0919 [M + H]^+^/0.4/181, 163	359.0770 [M − H]^−^/−0.4/197, 179, 161	Salvianolic acid A
29	10.78	C_44_H_86_O_14_	495.1225 [M + H]^+^/−2.2/297	493.1120 [M − H]^−^/−4.0/295	Not identify
30	11.19	C_65_H_106_O_32_	839.6083 [M + H]^+^/−0.8/661, 351	837.5897 [M − H]^−^/−5.7/791	Macranthoidin B ^a^
31	11.49	C_36_H_30_O_16_	1399.671 [M + H]^+^/−2.0/1021, 897, 751	Null	Salvianolic acid E
32	12.20	C_30_H_46_O_8_	719.1604 [M + H]^+^/−0.3/521, 323	717.1458 [M − H]^−^/−0.4/519	Neriifolin ^b^, [17]
33	13.24	C_48_H_72_O_21_	Null	579.3164 [M + COOH]^−^/−0.3/533, 515, 399	Licorice saponin A3
34	13.99	C_53_H_86_O_22_	985.4631 [M + H]^+^/−0.7/809,647,615	983.4493 [M − H]^−^/−0.1/821	Dipsacoside B ^a^
35	15.05	C_42_H_62_O_17_	1075.566 [M + H]^+^/−1.7/967, 863	Null	Licorice saponin G2
36	16.07	C_42_H_62_O_16_	839.4053 [M + H]^+^/−0.7/663, 487, 469, 451	837.3916 [M − H]^−^/0.3/351, 193	Glycyrrhizic acid ^a^

^a^ The identity were confirmed by comparing its t_R_, ESI-TOF-MS data with those of the reference substances. ^b^ Represented that compounds were identified with literatures. ^c^ Not detected.

**Table 2:** Linear-regression data, LODs, LOQs and recovery of the 14 constituents determined by HPLC-MS/MS.

Analyte	EIC Ions	Regression Equation	R^2^	Linear Range (μg/L)	LOD (μg/L)	LOQ (μg/L)	Repeatability RSD (%) (*n* = 6)		Stability RSD (%) (*n* = 6)	Standard Addition Recovery ^a^ (%) Mean ± SD (*n* = 6)
							Intra-Day	Inter-Day		
**4**	153.1	y = 8599.2x + 128.19	0.9995	36~900	6	30	1.56	1.85	2.35	96.75 ± 1.57
**5**	137.1	y = 37255x + 254.11	0.9992	2.1~315	1	5	2.14	2.45	2.68	99.57 ± 2.77
**9**	353.1	y = 15805x + 137.776	0.9994	14.4~1440	5	10	2.63	3.85	1.46	101.17 ± 2.53
**10**	179.1	y = 20031x + 1383.5	0.9995	30~3000	20	30	2.98	3.42	4.87	103.51 ± 1.01
**11**	609.1	y = 13077x + 369.10	0.9996	30~3000	0.5	1	3.15	3.61	3.56	97.12 ± 1.23
**16**	417.2	y = 52008x + 417.68	0.9995	2.4~480	0.2	0.5	2.81	2.65	2.15	103.90 ± 1.20
**18**	763.2	y = 24665x + 78.310	0.9998	4.08~408	1	4	2.36	4.75	4.42	101.28 ± 3.63
**19**	593.1	y = 13603x + 559.91	0.9993	48~2400	0.5	1	2.25	3.47	2.49	99.24 ± 3.36
**20**	623.2	y = 16622x + 33.444	0.9996	3.6~150	0.5	1	1.93	2.38	3.52	97.96 ± 2.20
**22**	447.1	y = 27716x + 277.58	0.9997	6~900	0.2	1	3.19	4.28	1.94	98.53 ± 3.21
**23**	515.1	y = 7453.0x − 121.38	0.9994	36~2700	10	20	1.95	2.22	2.73	97.77 ± 1.65
**27**	359.1	y = 12867x − 23.768	0.9996	12~1200	5	10	2.48	3.42	3.72	97.57 ± 1.19
**30**	1397.7	y = 557.36x + 1.2437	0.9994	10~120	1	10	1.46	2.17	3.18	102.94 ± 3.16
**34**	1073.6	y = 2184.1x + 3.8435	0.9992	3.6~120	1	2	2.85	3.14	2.65	105.78 ± 4.12

^a^ The data are presented as the average of six determinations, where standard addition recovery (%) = 100 × (amount found-original)/amount spiked.

**Table 3:** The contents of 17 analytes in 16 batches of WLJHT by HPLC-MS/MS and HPLC-ELSD (μg/mL).

Analyte	W141009	W141011	W141012	W141013	W141015	W141021	W141023	W141024	W141025	W141026	W141028	W141030	W141117	W141118	W141119	W141120
**4**	0.76	0.85	0.79	0.80	0.86	0.77	0.81	0.80	0.78	0.77	0.79	0.82	0.80	0.81	0.81	0.80
**5**	0.62	0.64	0.61	0.60	0.65	0.63	0.56	0.63	0.57	0.57	0.58	0.57	0.57	0.57	0.56	0.54
**9**	3.25	3.49	3.28	4.05	3.34	3.63	3.53	3.51	3.23	4.03	3.54	3.42	3.51	3.50	3.42	3.26
**10**	0.54	0.53	0.55	0.56	0.56	0.62	0.58	0.56	0.51	0.55	0.52	0.54	0.46	0.48	0.49	0.43
**11**	0.70	0.68	0.64	0.71	0.65	0.66	0.65	0.64	0.64	0.64	0.71	0.68	0.70	0.71	0.68	0.68
**16**	3.46	3.72	3.55	3.29	3.61	3.34	3.14	3.24	3.13	3.23	3.54	3.23	3.26	3.14	3.06	2.96
**18**	0.05	0.04	0.04	0.05	0.04	0.04	0.03	0.04	0.05	0.05	0.04	0.04	0.04	0.05	0.04	0.04
**19**	0.66	0.64	0.58	0.66	0.58	0.63	0.64	0.62	0.61	0.57	0.63	0.64	0.73	0.68	0.62	0.63
**20**	1.23	1.26	1.13	1.24	1.15	1.16	1.18	1.24	1.16	1.15	1.32	1.26	1.42	1.23	1.14	1.25
**22**	1.45	1.43	1.36	1.54	1.37	1.56	1.53	1.58	1.56	1.57	1.46	1.51	1.57	1.69	1.56	1.41
**23**	2.36	2.23	2.22	2.31	2.24	2.23	2.34	2.13	2.20	2.26	2.12	2.10	2.27	2.26	2.11	1.87
**27**	9.65	9.13	9.11	10.28	9.22	9.54	9.58	9.23	10.12	10.07	9.85	9.35	9.22	10.15	9.49	8.78
**30**	0.05	0.03	0.03	0.07	0.08	0.08	0.08	0.04	0.06	0.06	0.04	0.05	0.06	0.06	0.03	0.03
**34**	0.03	0.01	0.04	0.02	0.02	0.02	0.02	0.01	0.01	0.02	0.02	0.01	0.02	0.01	0.03	0.02
**Sub-total**	24.81	24.68	23.93	26.18	24.37	24.91	24.67	24.27	24.63	25.54	25.16	24.22	24.63	25.34	24.04	22.79
**Fructose ^a^**	5.87	7.14	6.69	6.06	6.52	5.96	6.32	5.95	6.66	7.55	6.52	5.99	6.31	6.51	7.17	8.39
**Glucose ^a^**	7.44	8.20	7.50	7.67	7.56	8.29	8.32	8.11	7.65	7.44	8.44	7.20	8.23	7.96	7.25	7.69
**Sucrose ^a^**	60.23	63.60	62.42	61.48	66.98	66.33	62.14	62.99	64.05	64.48	66.04	61.21	62.41	64.83	61.44	63.82
**Dry weight ^a^**	86.46	87.68	91.43	95.71	91.69	92.60	91.52	88.08	92.84	88.78	94.17	87.03	93.96	98.05	91.68	90.84
**Content (%)**	85.09	90.06	83.82	78.61	88.43	87.05	83.92	87.50	84.43	89.54	86.04	85.52	81.92	80.90	82.77	87.98

^a^ The unit of weight was mg/mL.

## Supplementary Materials

The following are available online.
