# Supplementary material for: Qualitative and Quantitative Analysis of the Major Constituents in WLJ Herbal Tea Using Multiple Chromatographic Techniques
Source: Molecules. 2018 Oct 12;23(10):2623. doi: 10.3390/molecules23102623 (PMC6222873; doi:10.3390/molecules23102623)
Supplement: Supplementary file 1 [file molecules-23-02623-s001.pdf]

Supplementary Information for

Comprehensive Quantitative Analysis of WLJ Herbal Tea Using Multiple Chromatographic Technologies

Chaozhan Lin [a], Runjing Zhang [a], Yufeng Yao [a], Chenchen Zhu [a,*]

[a] Institute of Clinical Pharmacology, Guangzhou University of Chinese Medicine, No.12 Jichang Rd, Guangzhou 510405, P.R. China

[*] Corresponding authors. Tel.: +86 2036585303; fax: +86 2036588015.

E-mail addresses: zhuchenchen@vip.sina.com (C.-C. Zhu).

Contents

The positive/negative MS/MS spectra of the identified compounds from WLJHT.

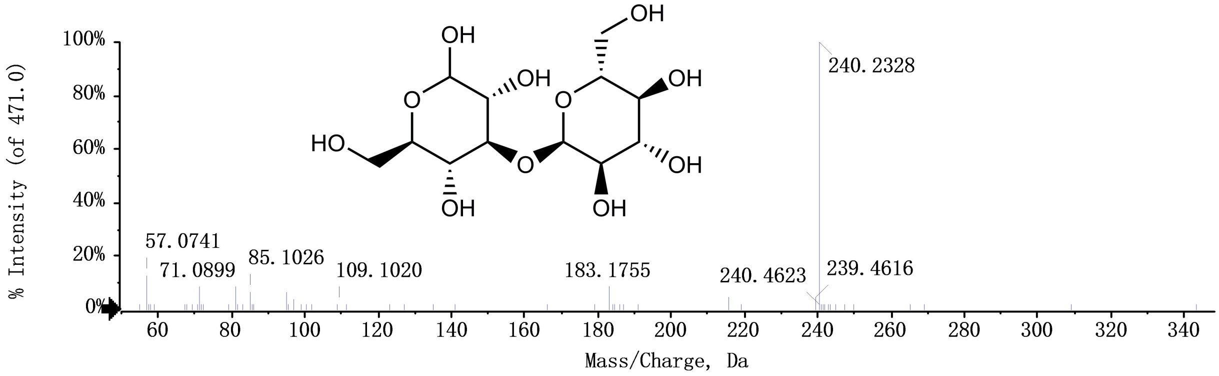

Figure S1The positive MS/MS spectra of compound 1

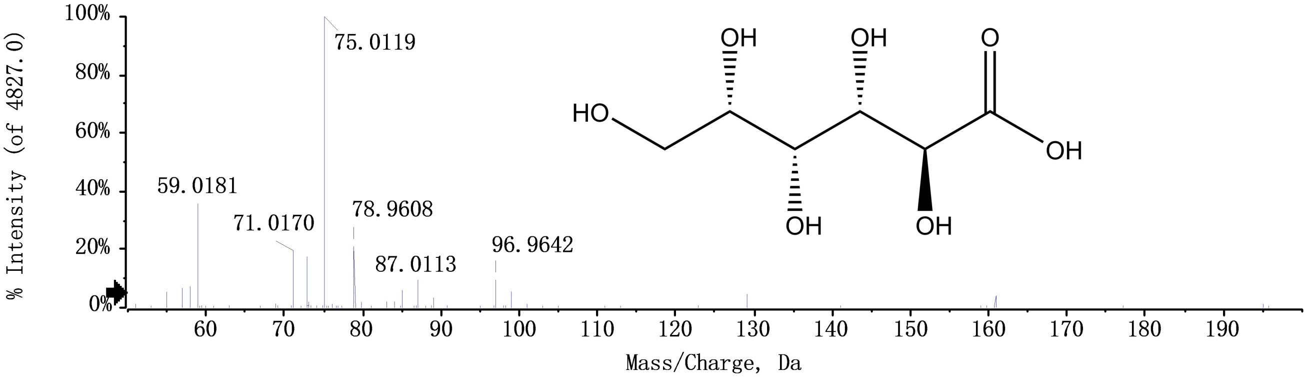

Figure S2 The negative MS/MS spectra of compound 2

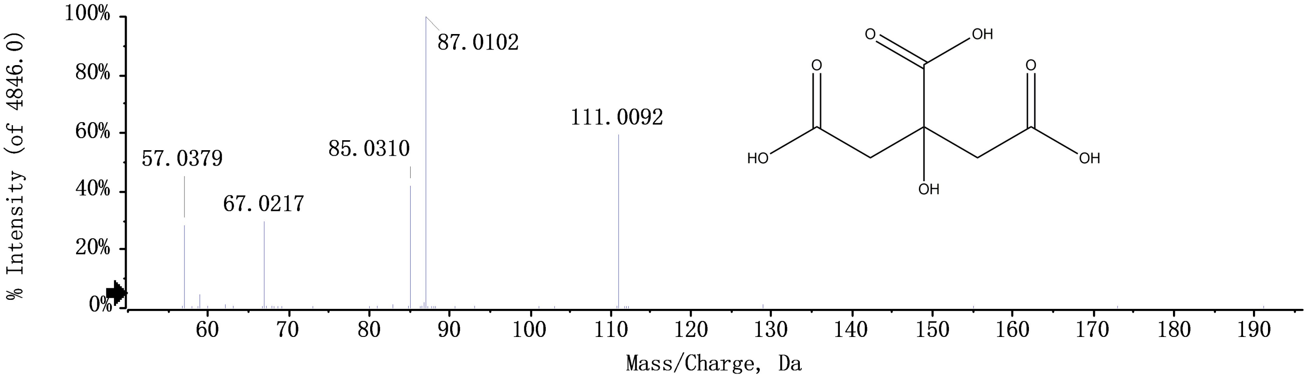

Figure S3 The negative MS/MS spectra of compound 3

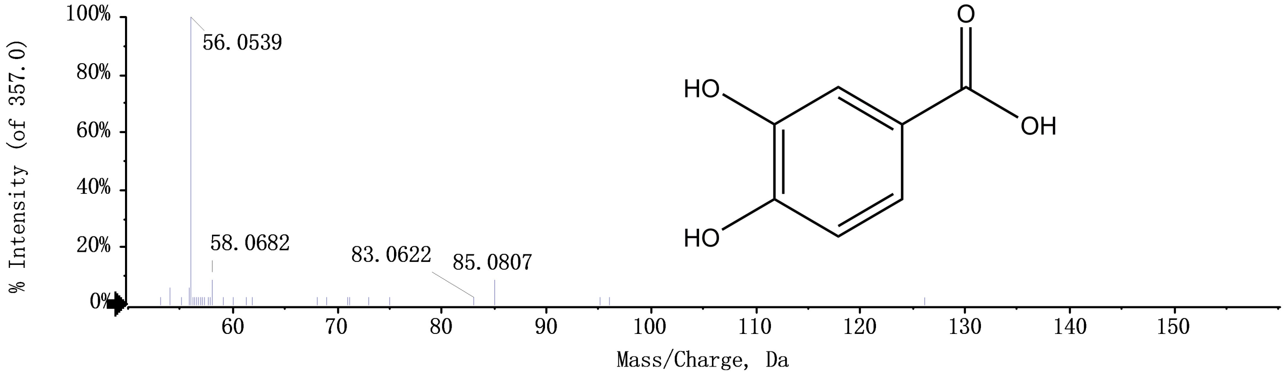

Figure S4A The positive MS/MS spectra of compound 4

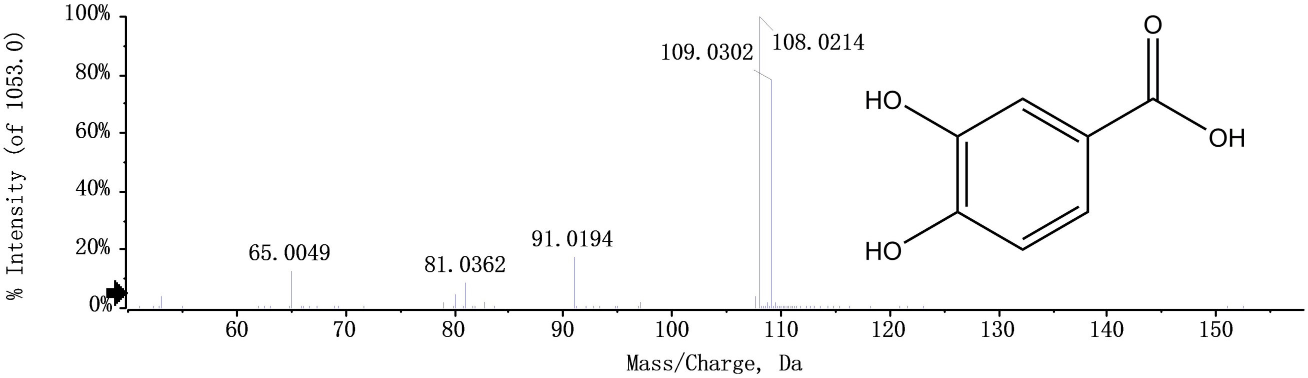

Figure S4B The negative MS/MS spectra of compound 4

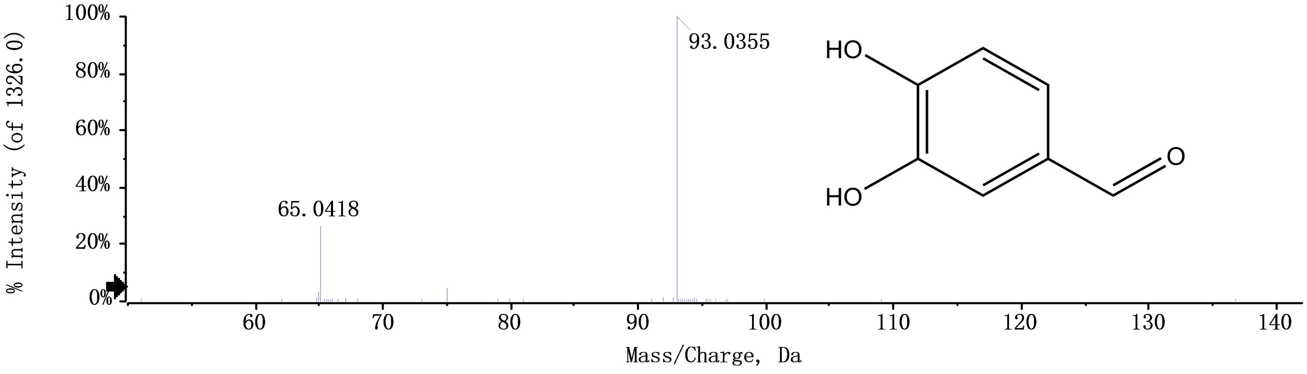

Figure S5 The negative MS/MS spectra of compound 5

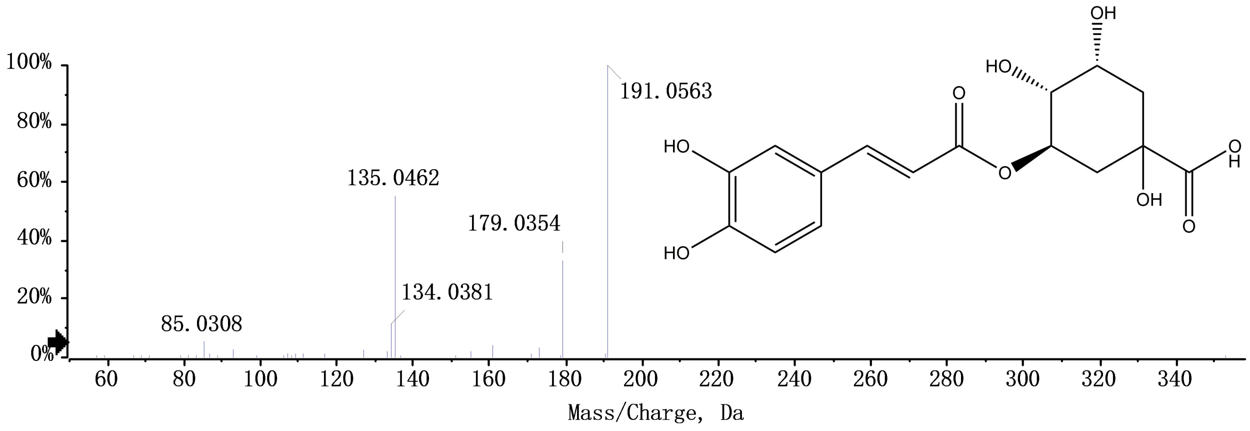

Figure S6 The negative MS/MS spectra of compound 6

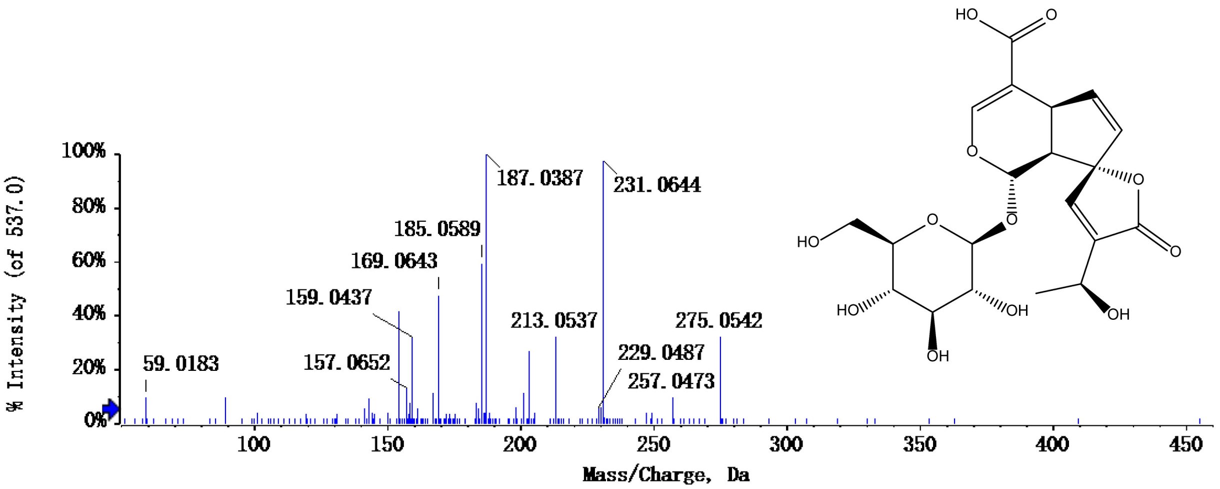

Figure S7The negative MS/MS spectra of compound 7

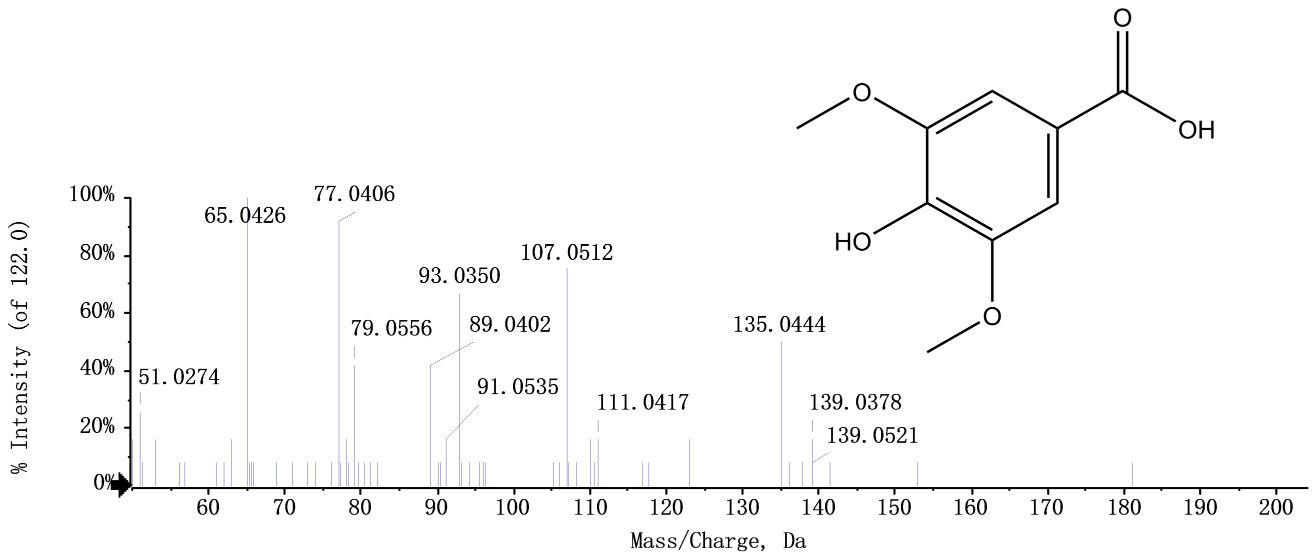

Figure S8A The positive MS/MS spectra of compound 8

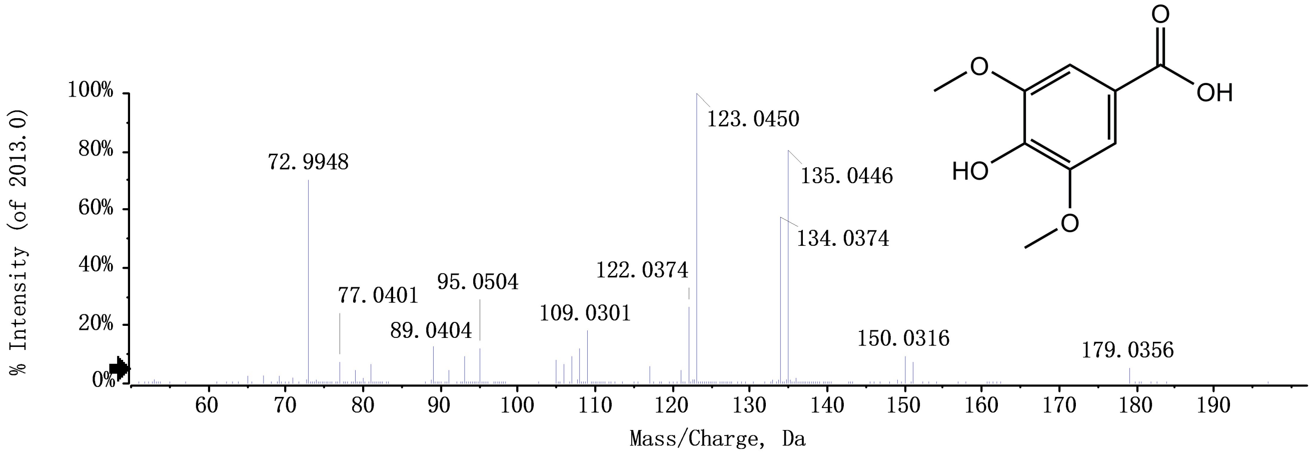

Figure S8B The negative MS/MS spectra of compound 8

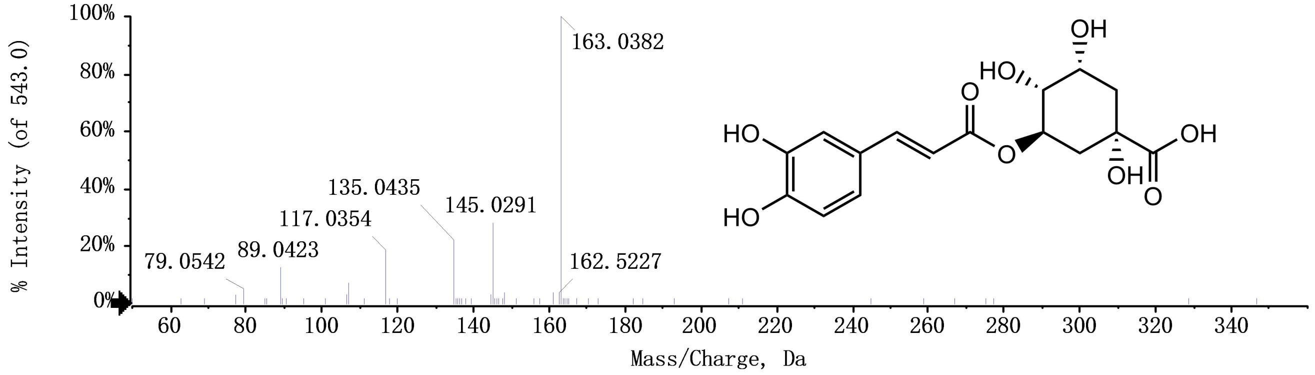

Figure S9A The positive MS/MS spectra of compound 9

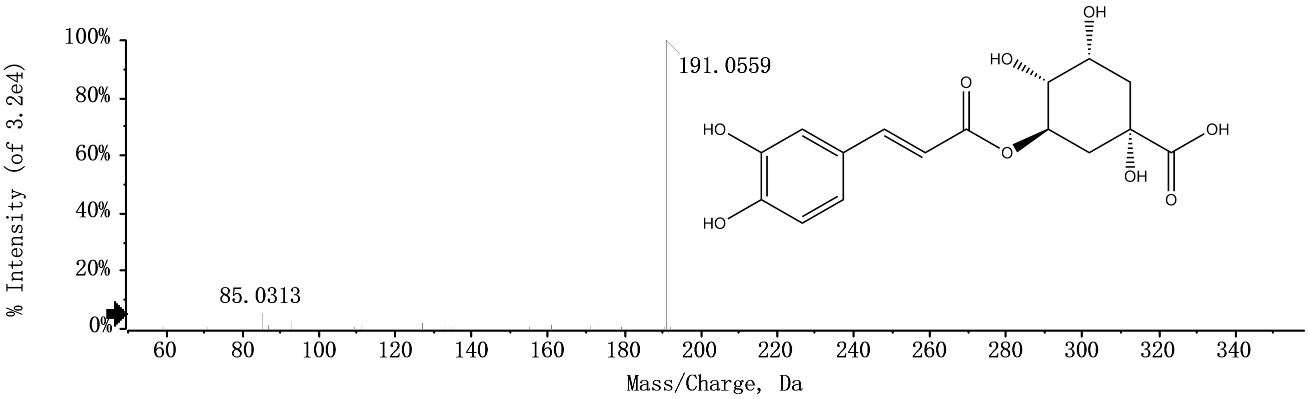

Figure S9B The negative MS/MS spectra of compound 9

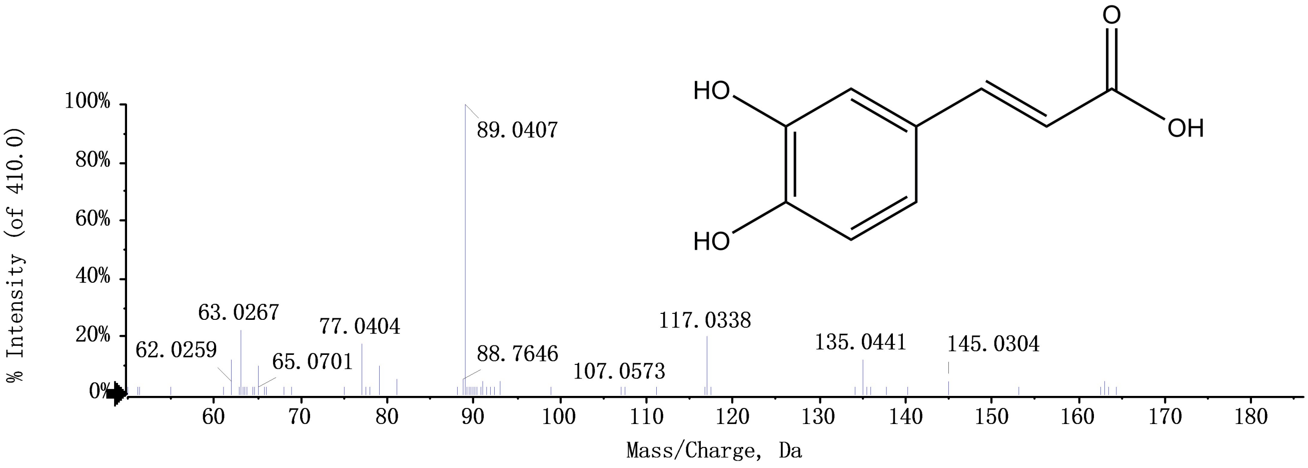

Figure S10A The positive MS/MS spectra of compound 10

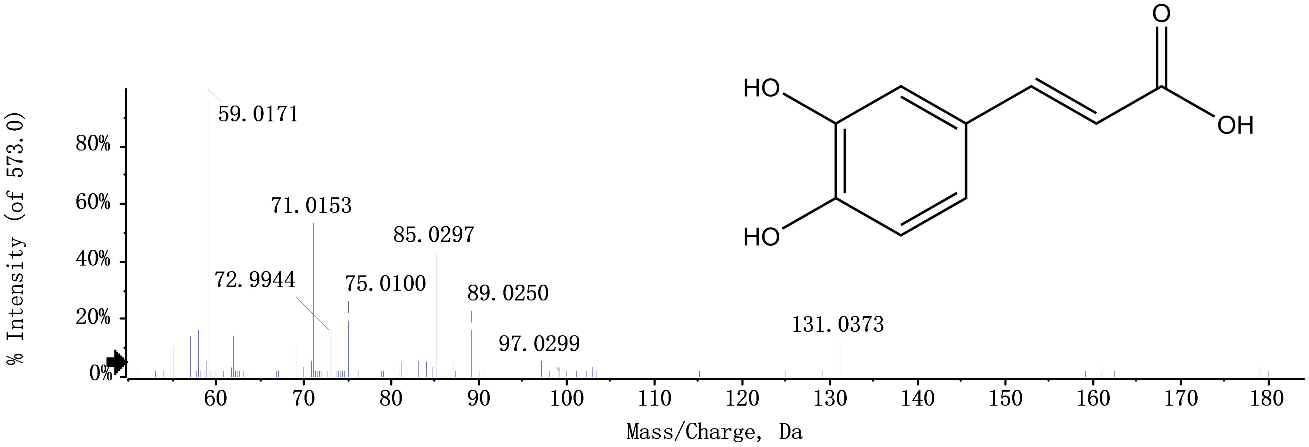

Figure S10B The negative MS/MS spectra of compound 10

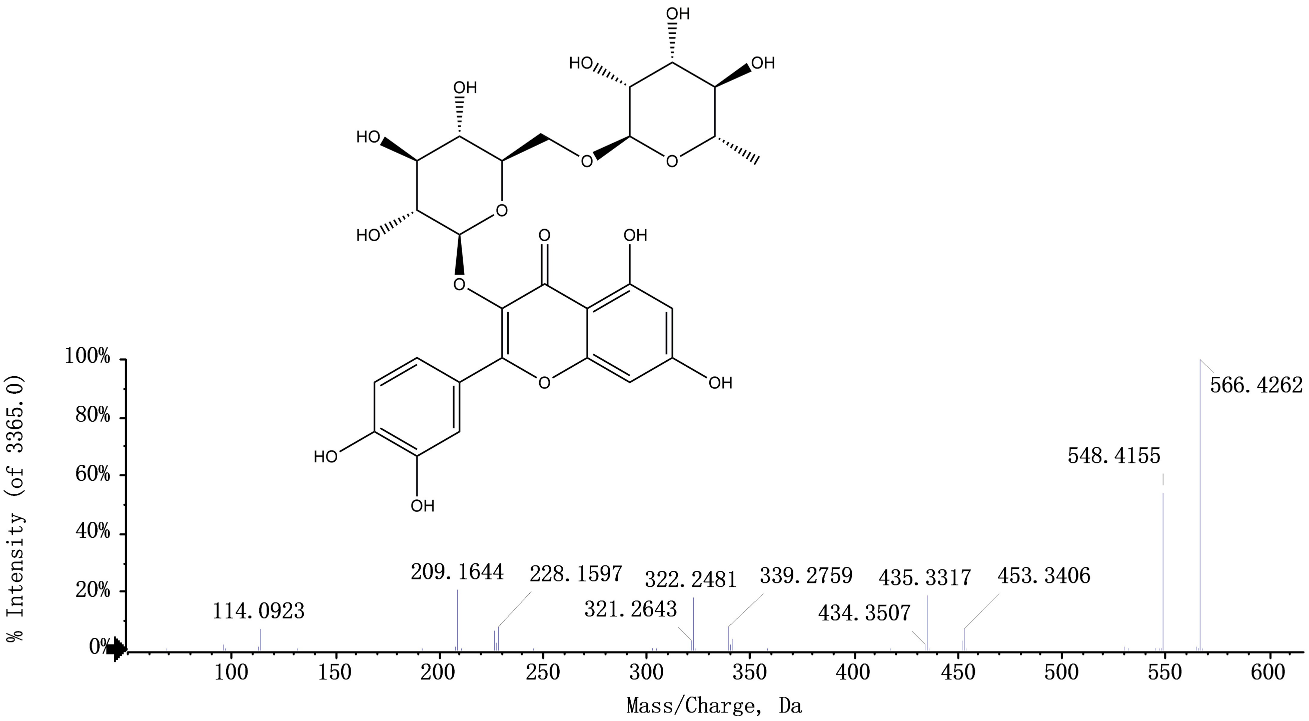

Figure S11 The positive MS/MS spectra of compound 11

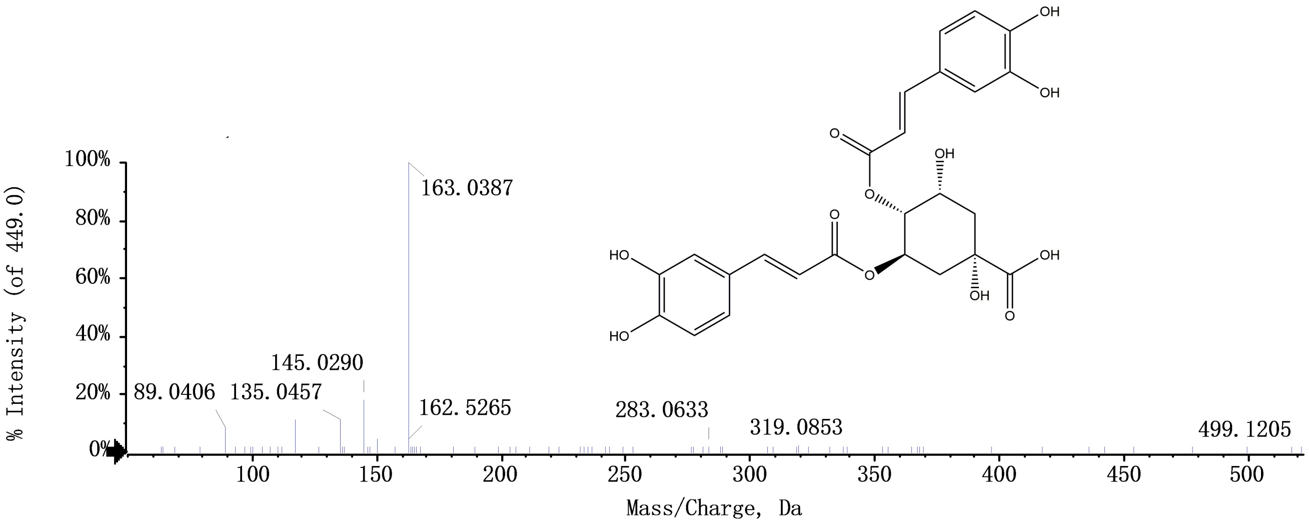

Figure S12A The positive MS/MS spectra of compound 12

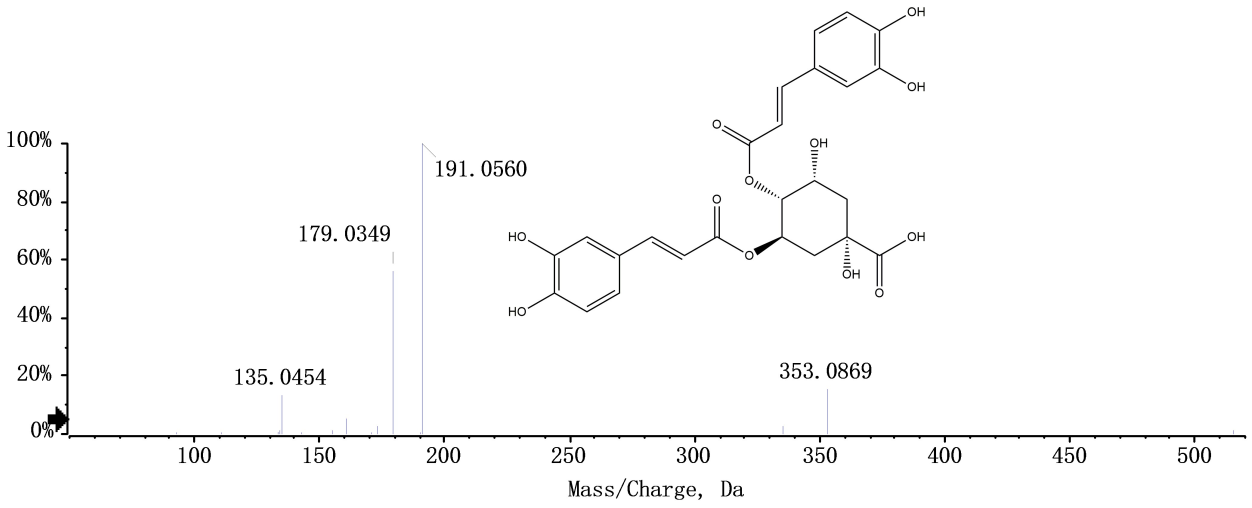

Figure S12B The negative MS/MS spectra of compound 12

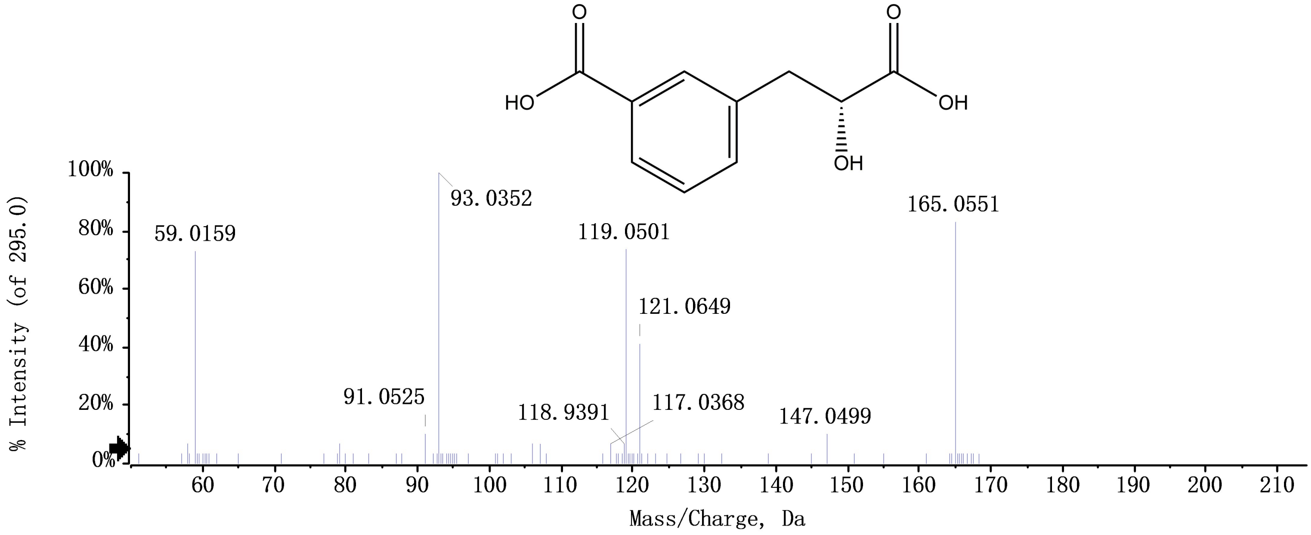

Figure S13 The negative MS/MS spectra of compound 13

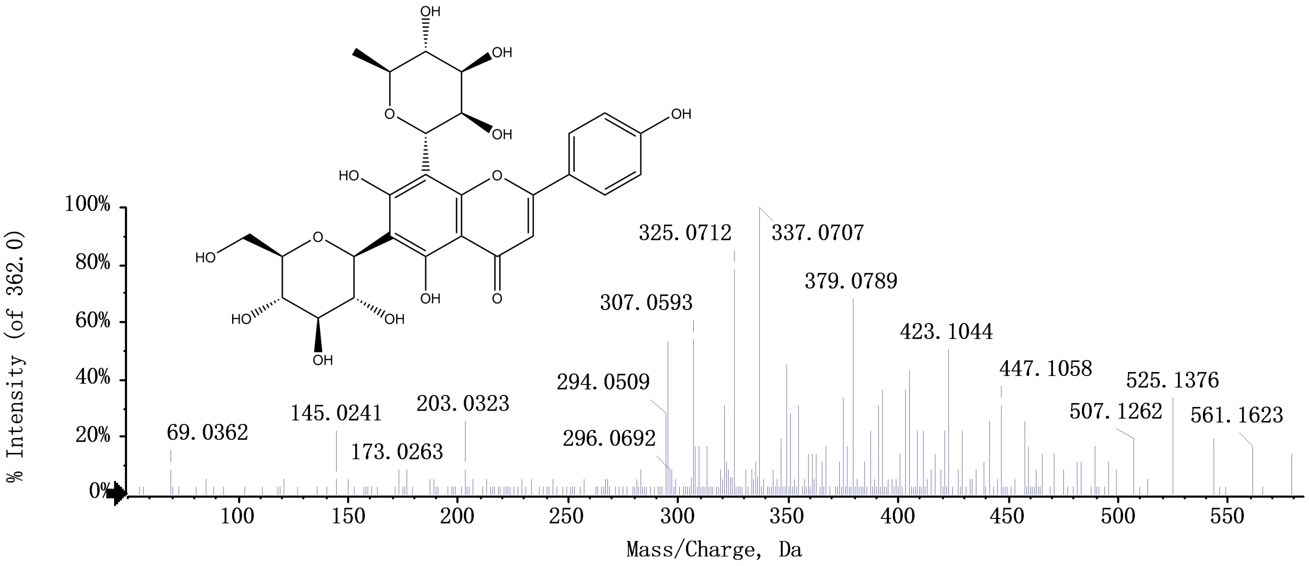

Figure S14A The positive MS/MS spectra of compound 14

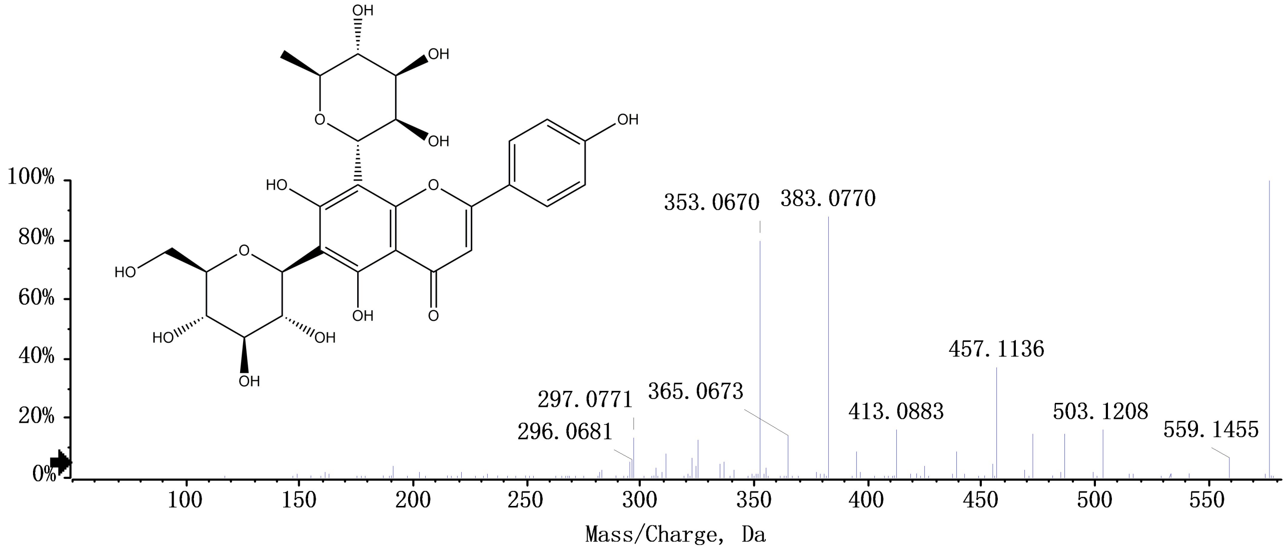

Figure S14B The negative MS/MS spectra of compound 14

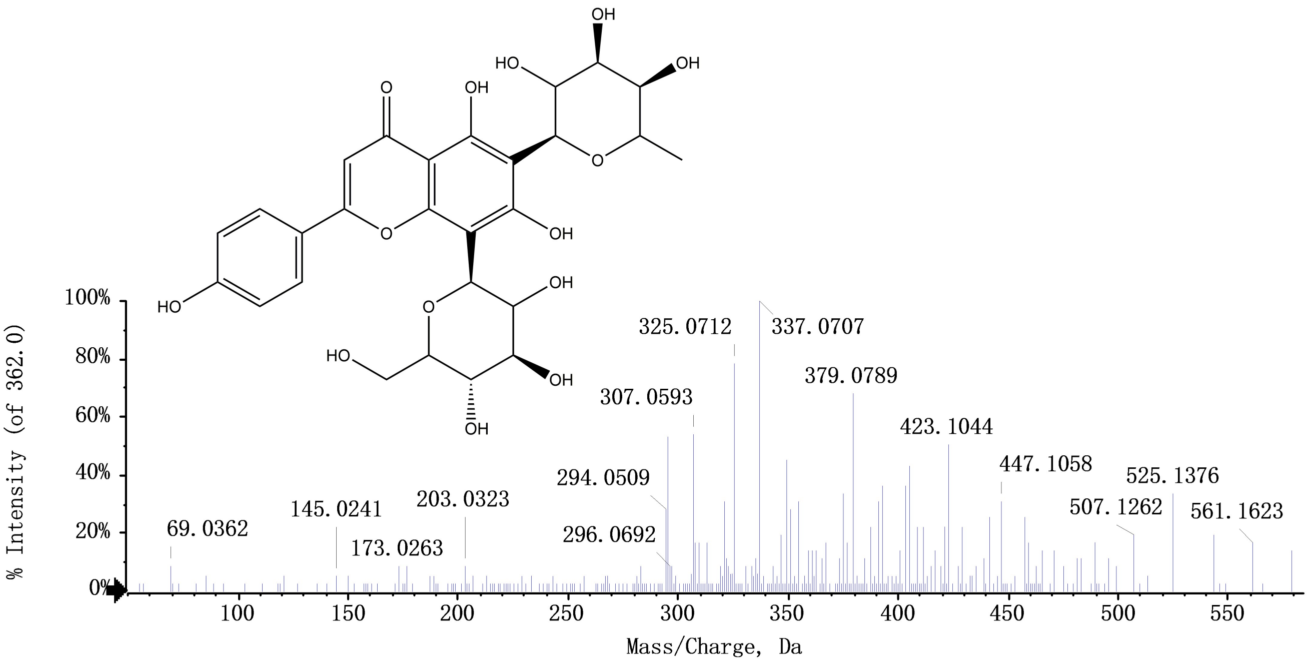

Figure S15A The positive MS/MS spectra of compound 15

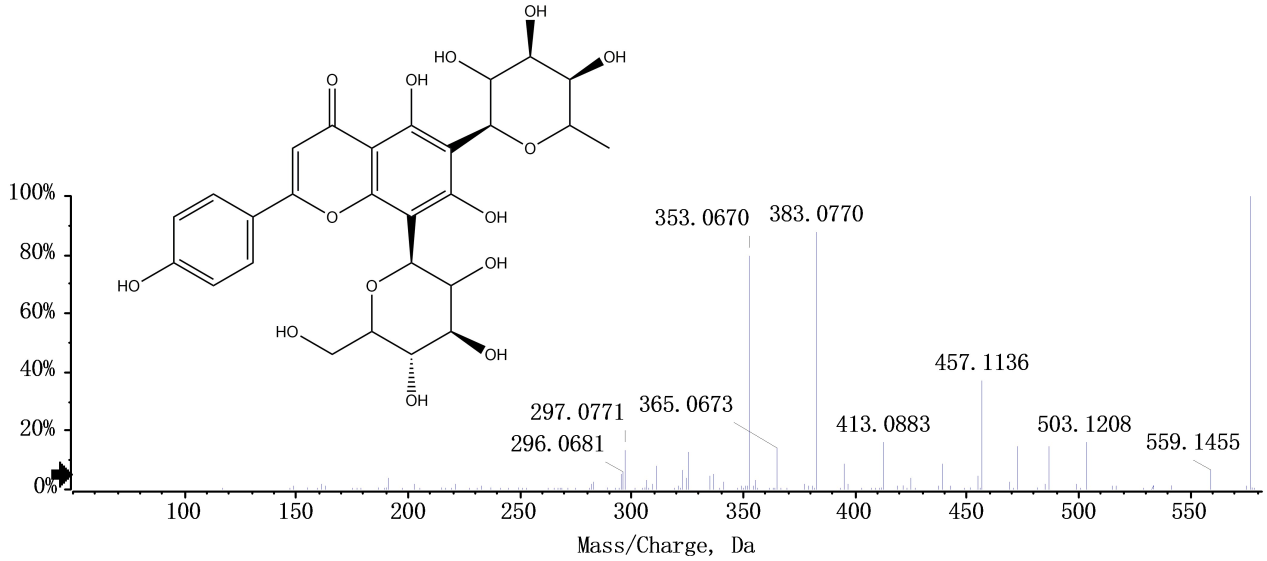

Figure S15B The negative MS/MS spectra of compound 15

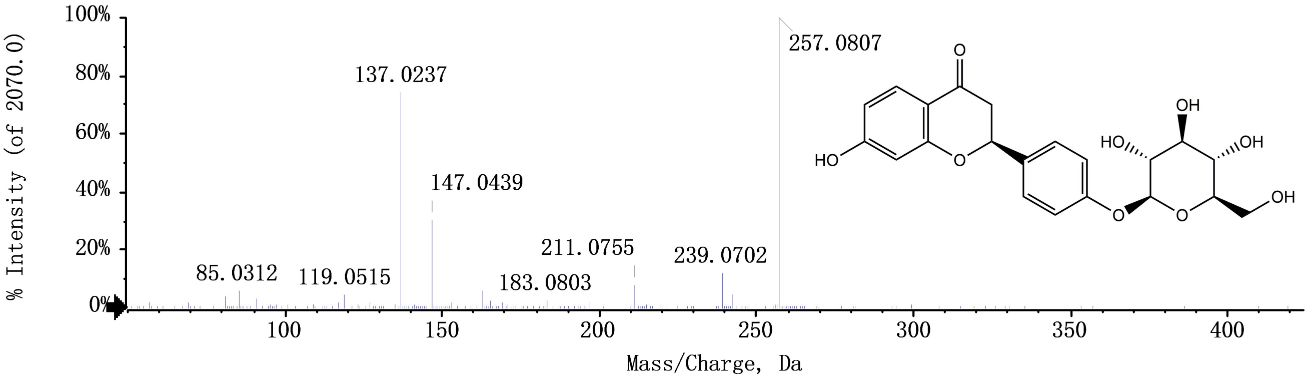

Figure S16A The positive MS/MS spectra of compound 16

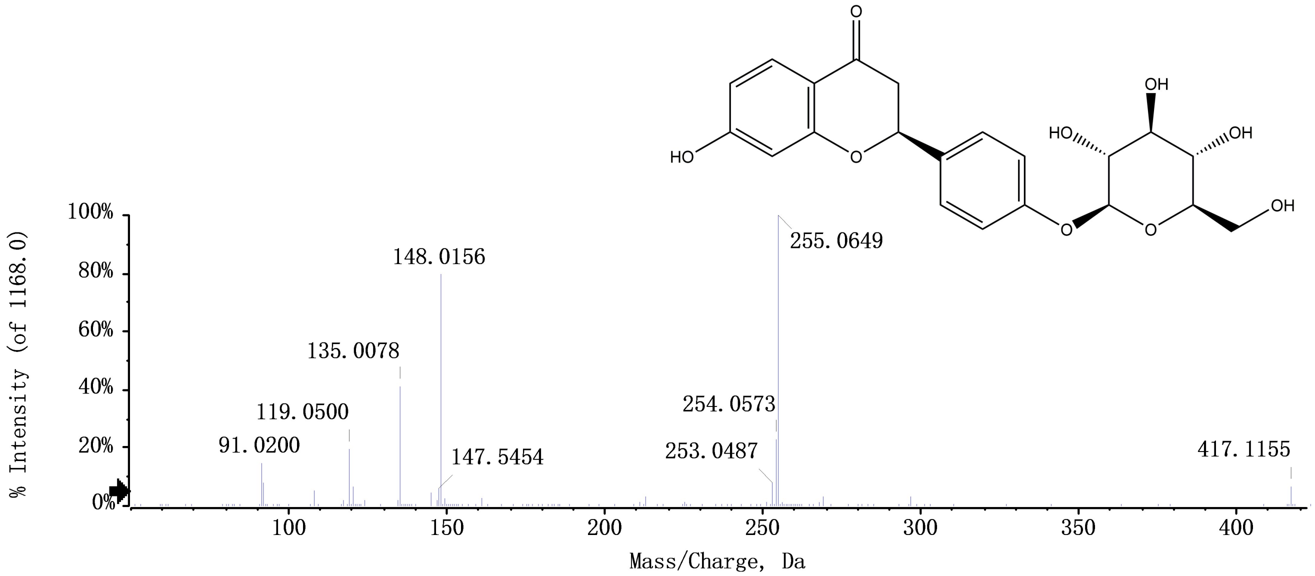

Figure S16B The negative MS/MS spectra of compound 16

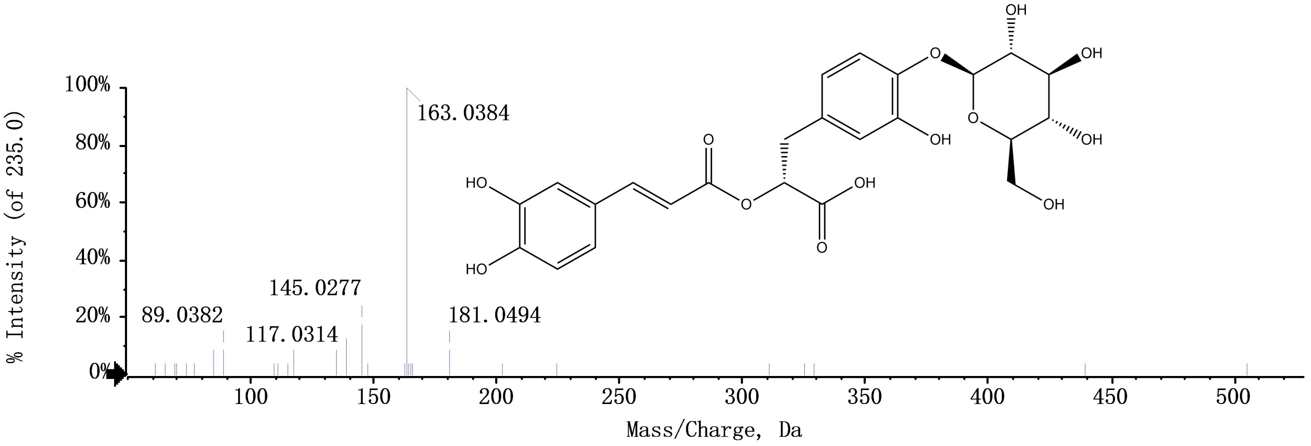

Figure S17A The positive MS/MS spectra of compound 17

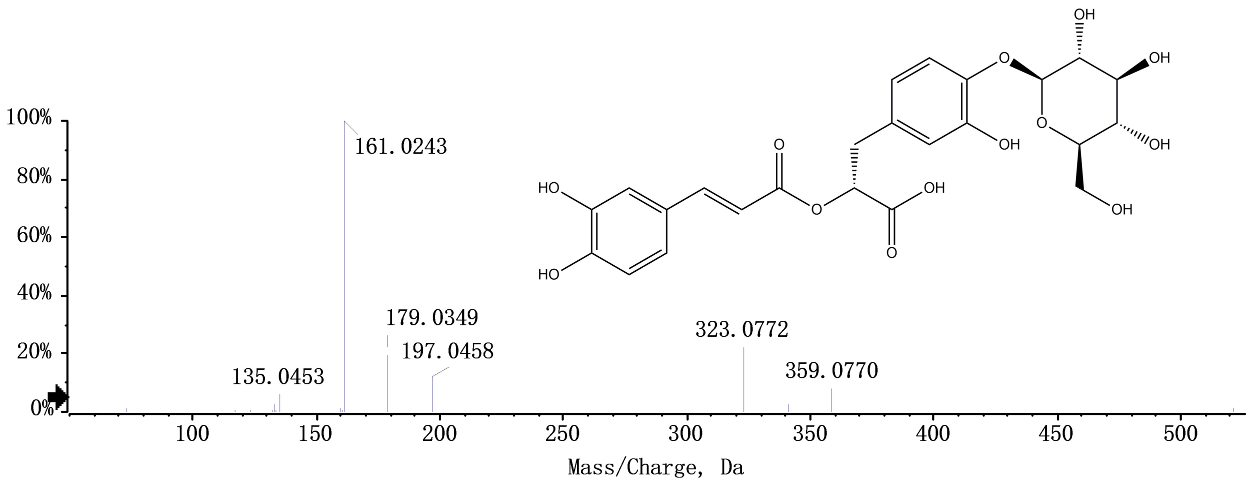

Figure S17B The negative MS/MS spectra of compound 17

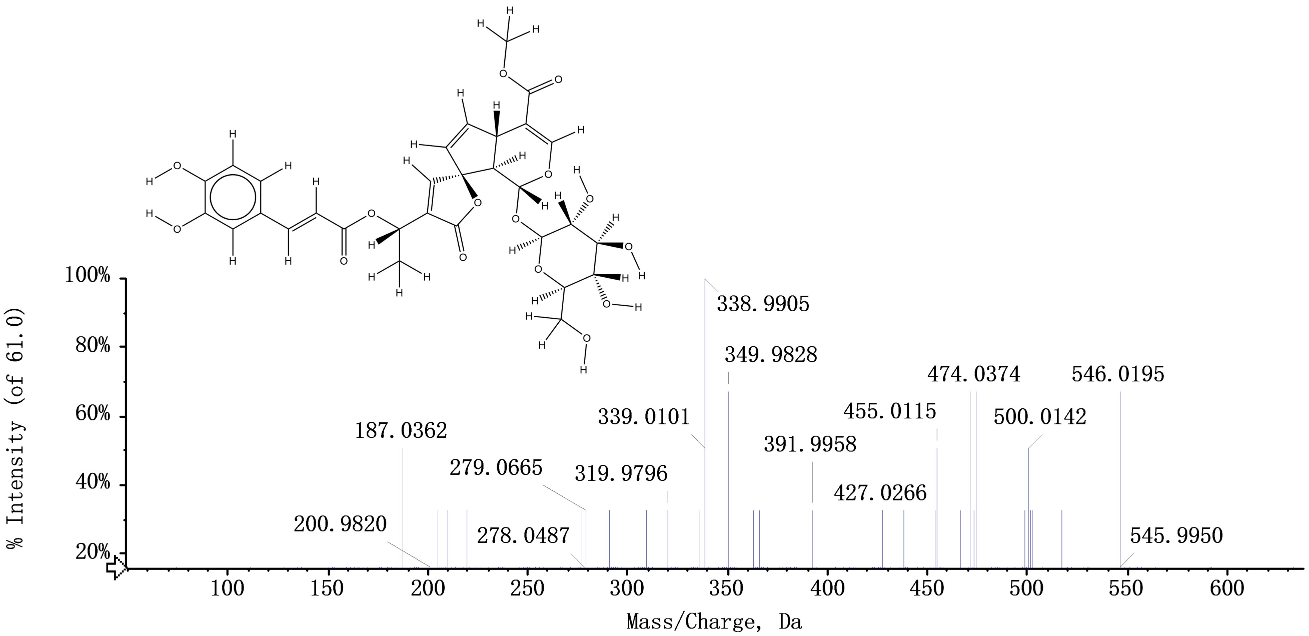

Figure S18 The positive MS/MS spectra of compound 18

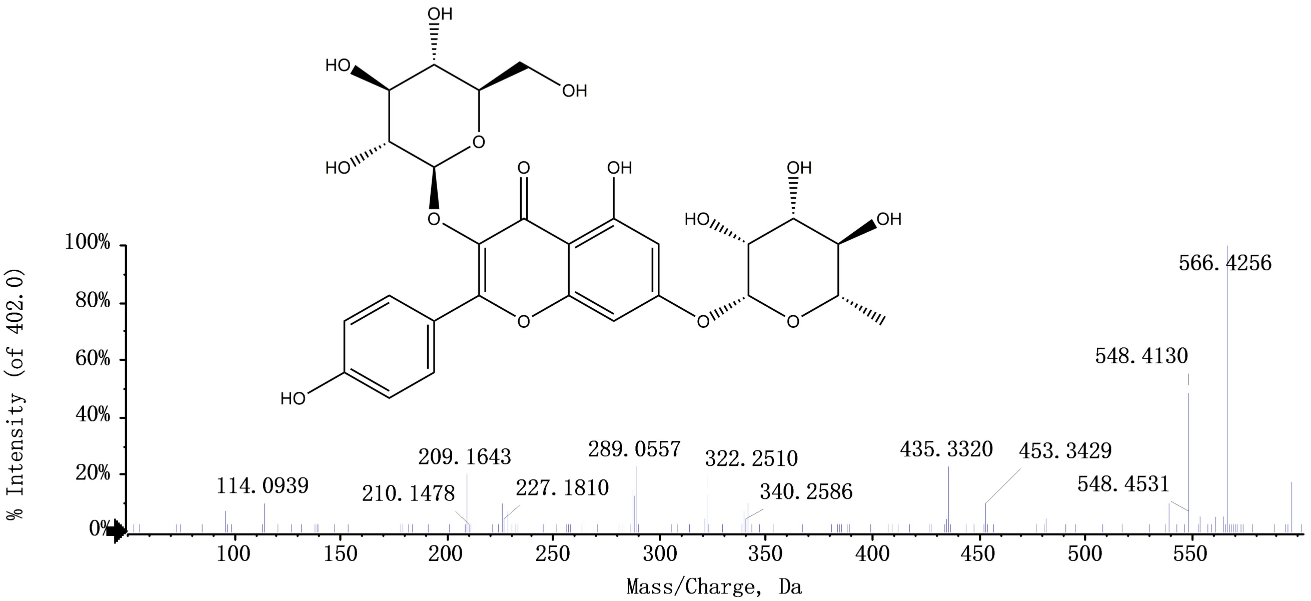

Figure S19 The positive MS/MS spectra of compound 19

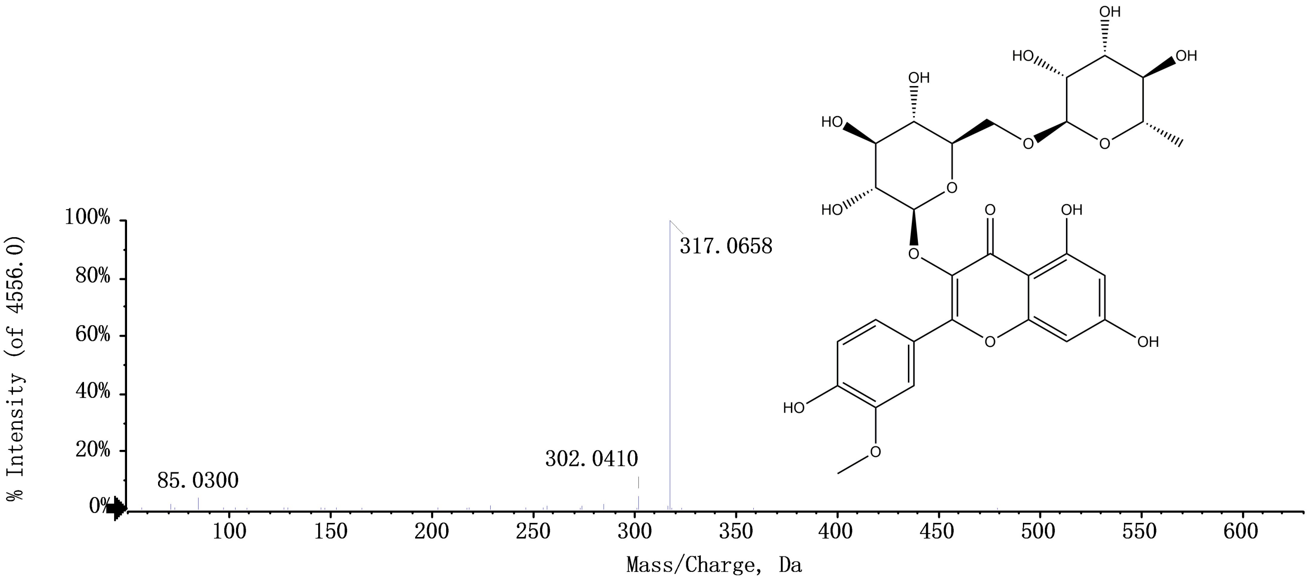

Figure S20A The positive MS/MS spectra of compound 20

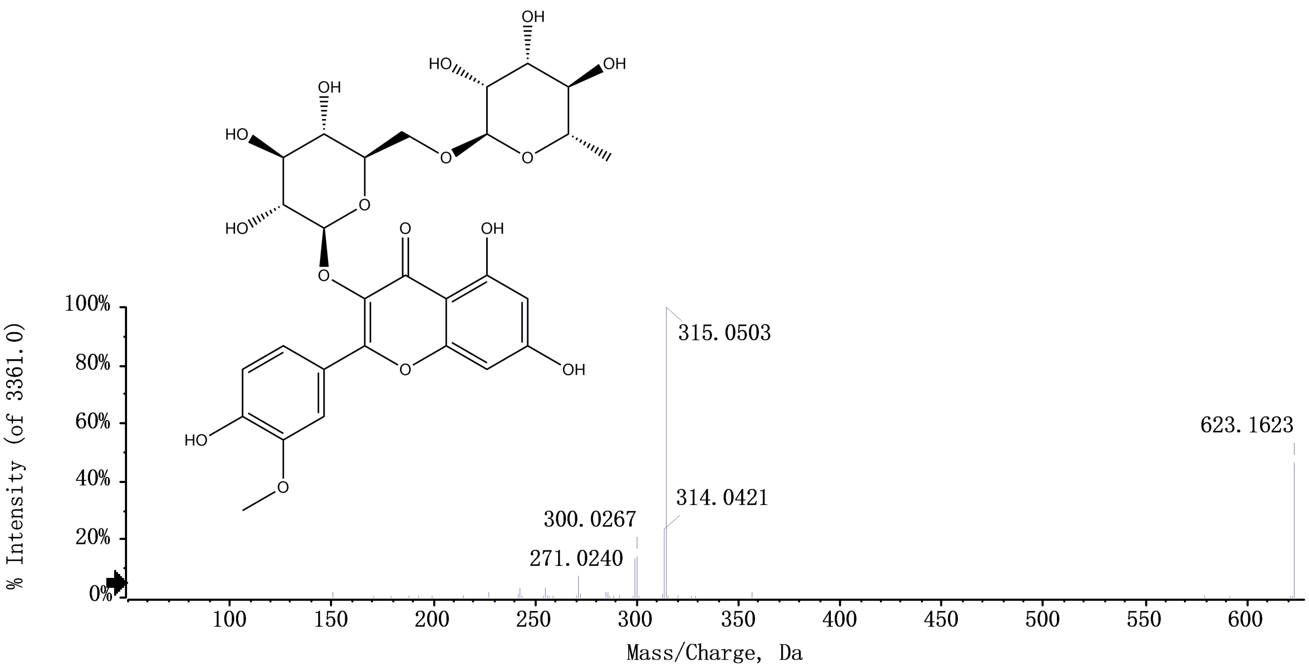

Figure S20B The negative MS/MS spectra of compound 20

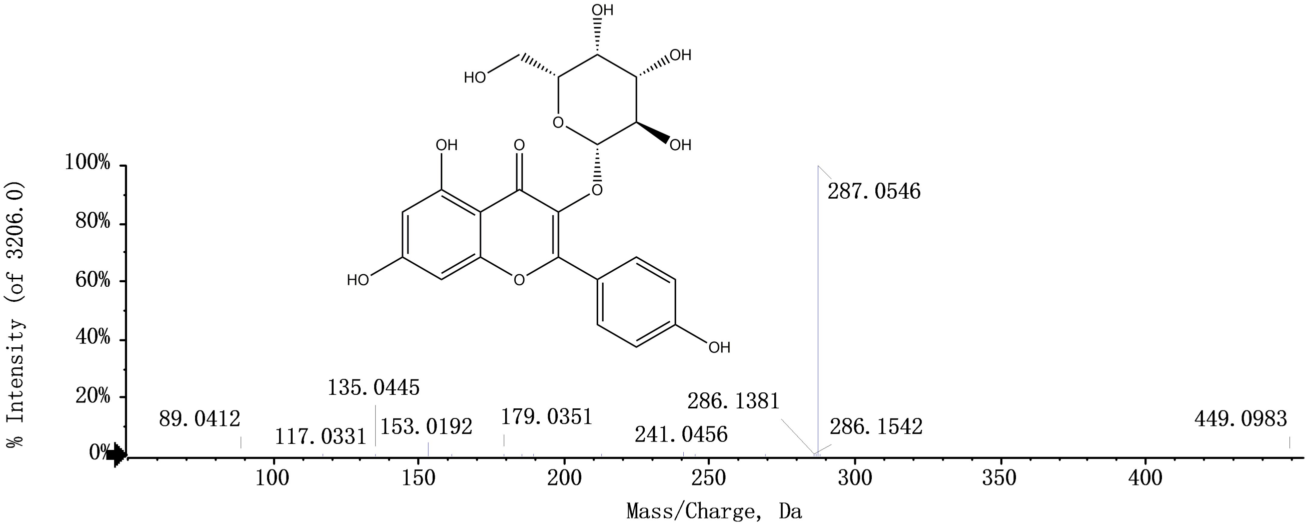

Figure S22A The positive MS/MS spectra of compound 22

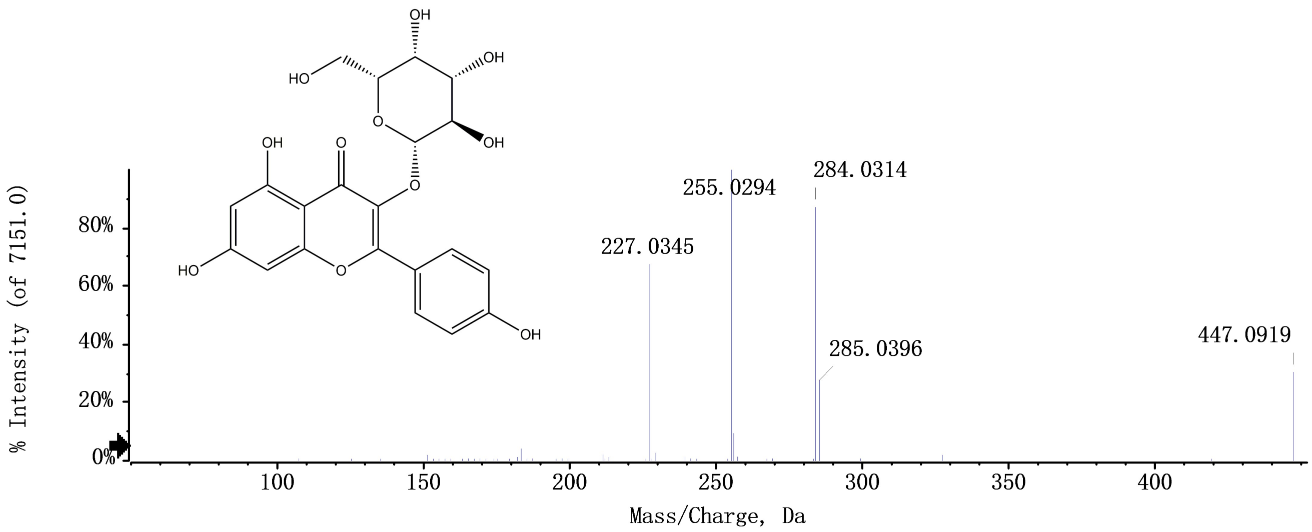

Figure S22B The negative MS/MS spectra of compound 22

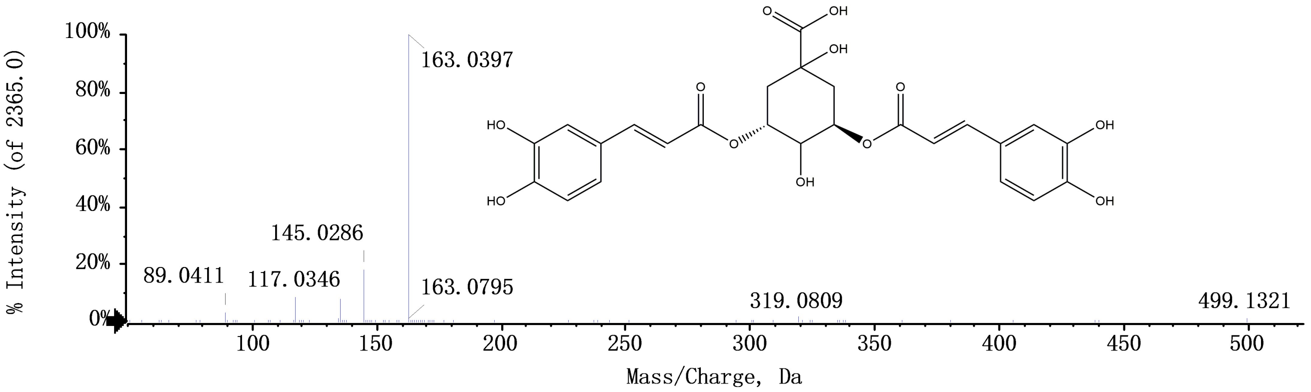

Figure S23A The positive MS/MS spectra of compound 23

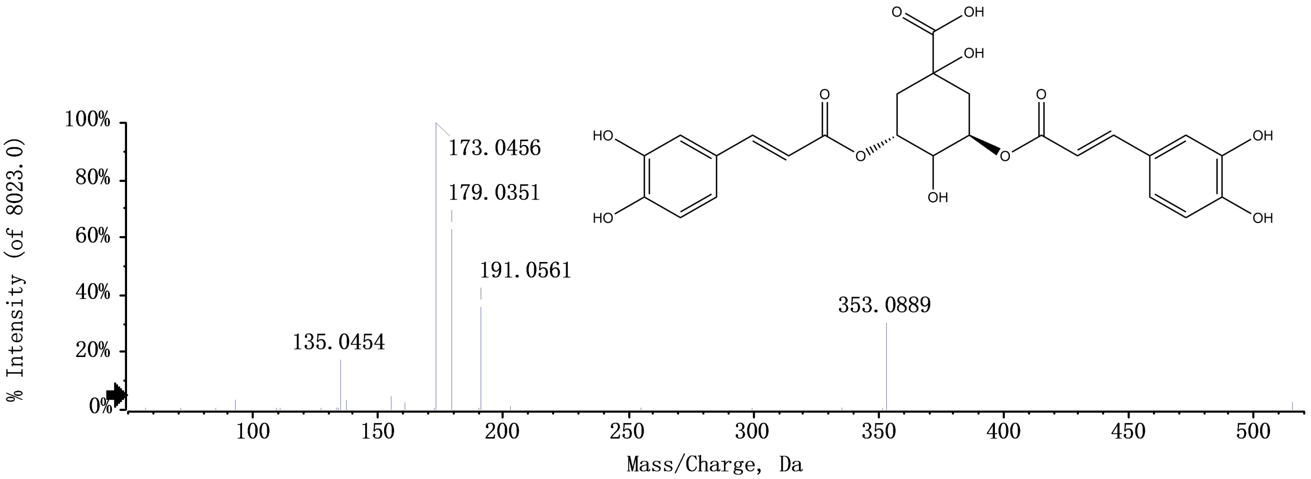

Figure S23B The negative MS/MS spectra of compound 23

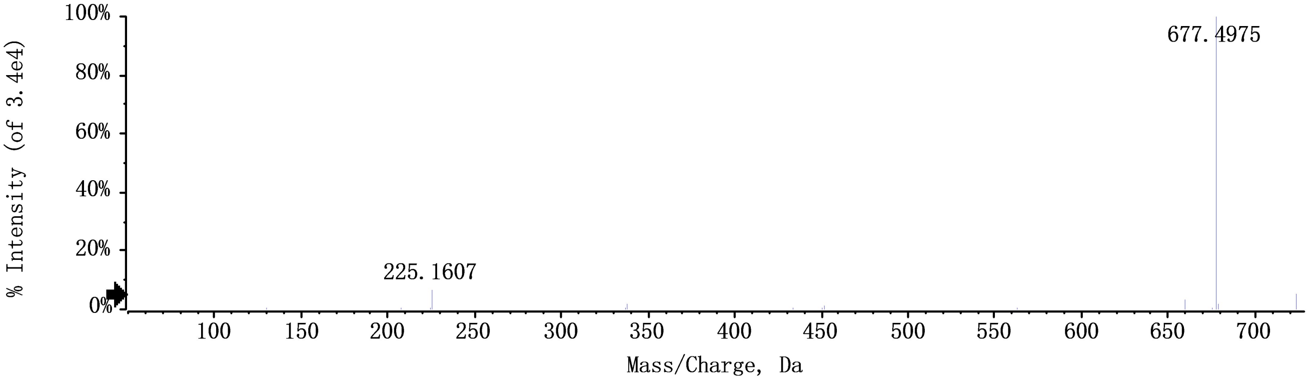

Figure S25 The negative MS/MS spectra of compound 25

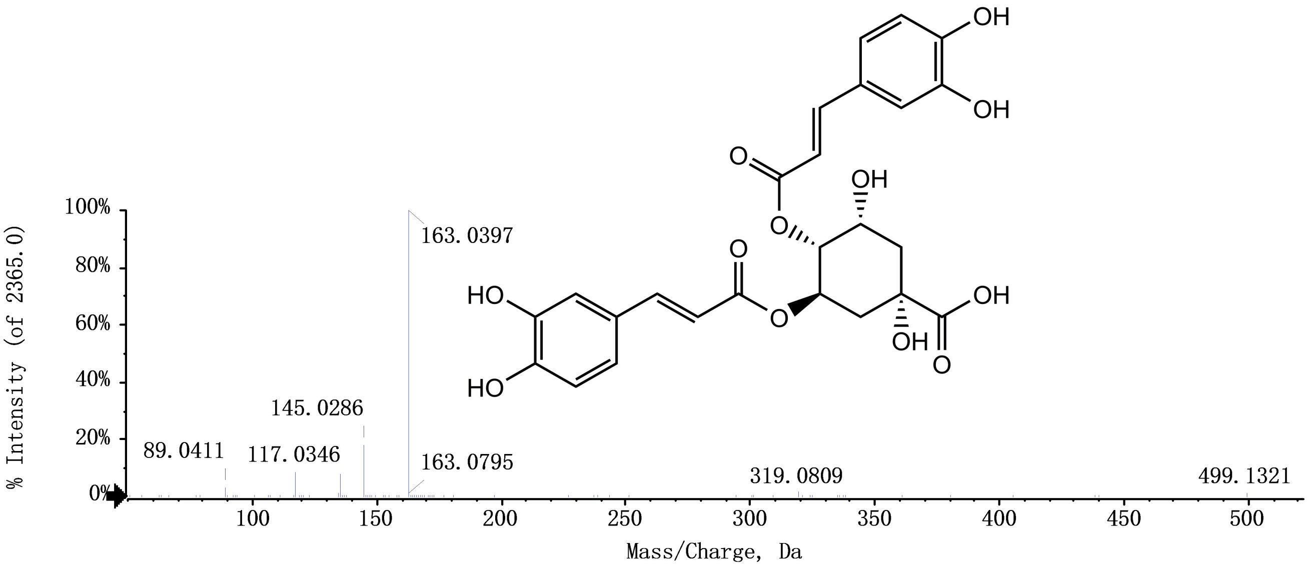

Figure S26 The positive MS/MS spectra of compound 26

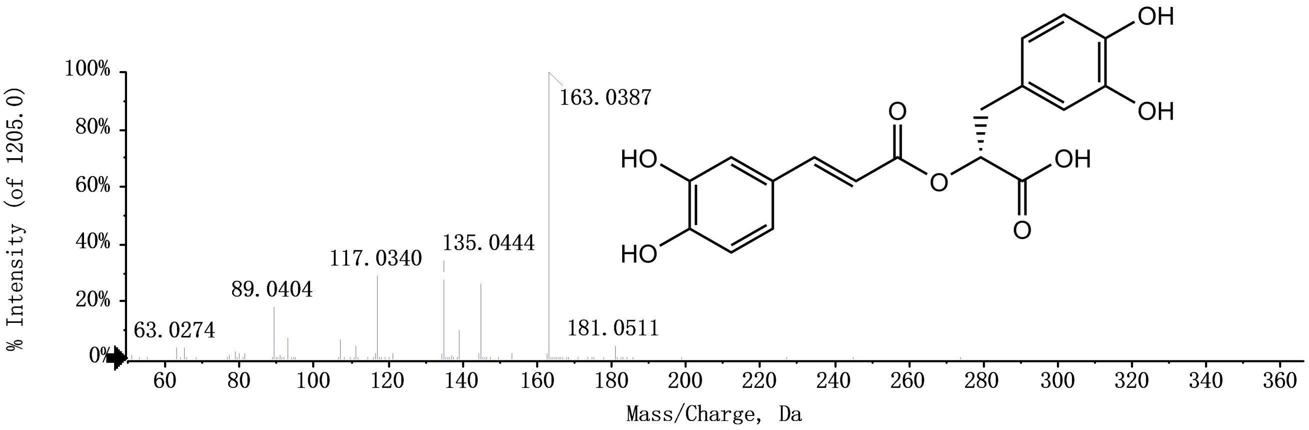

Figure S27A The positive MS/MS spectra of compound 27

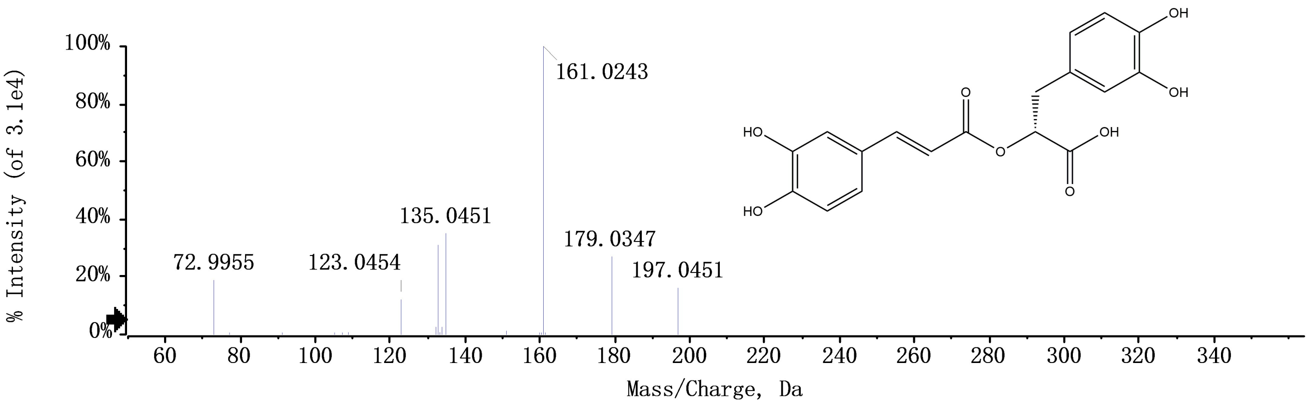

Figure S27B The negative MS/MS spectra of compound 27

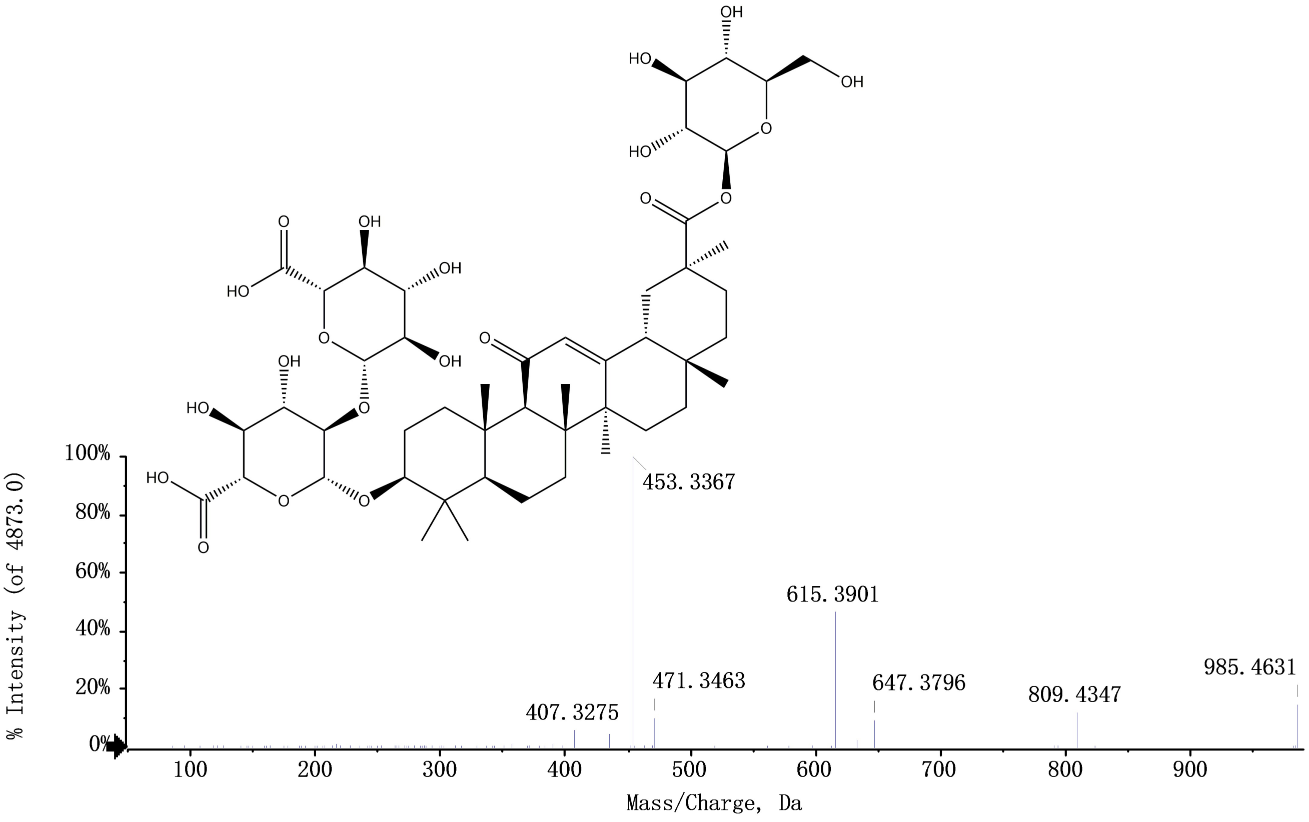

Figure S33A The positive MS/MS spectra of compound 33

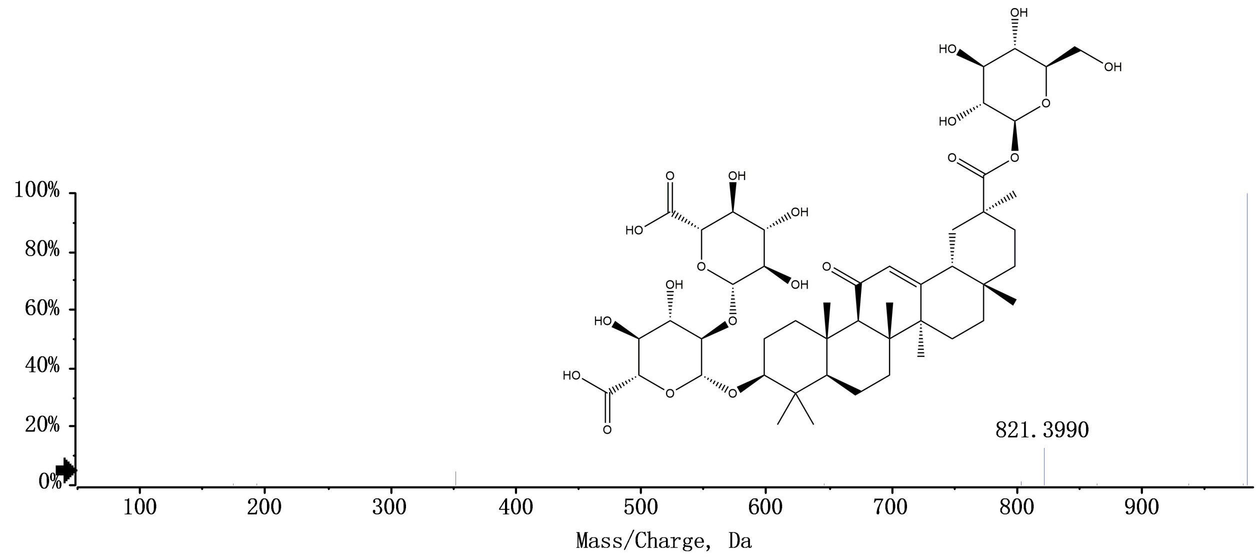

Figure S33B The negative MS/MS spectra of compound 33

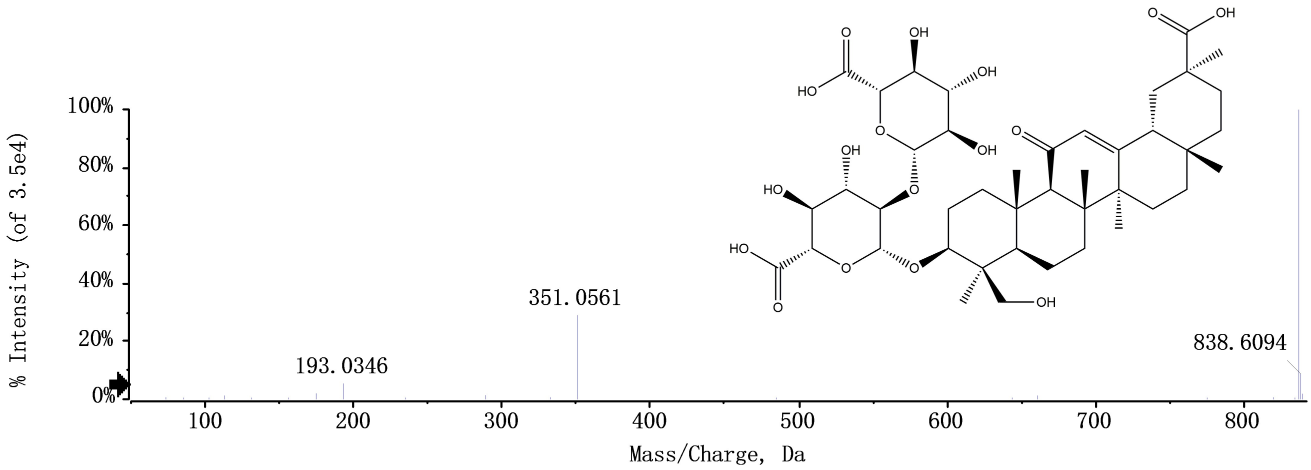

Figure S35 The negative MS/MS spectra of compound 35

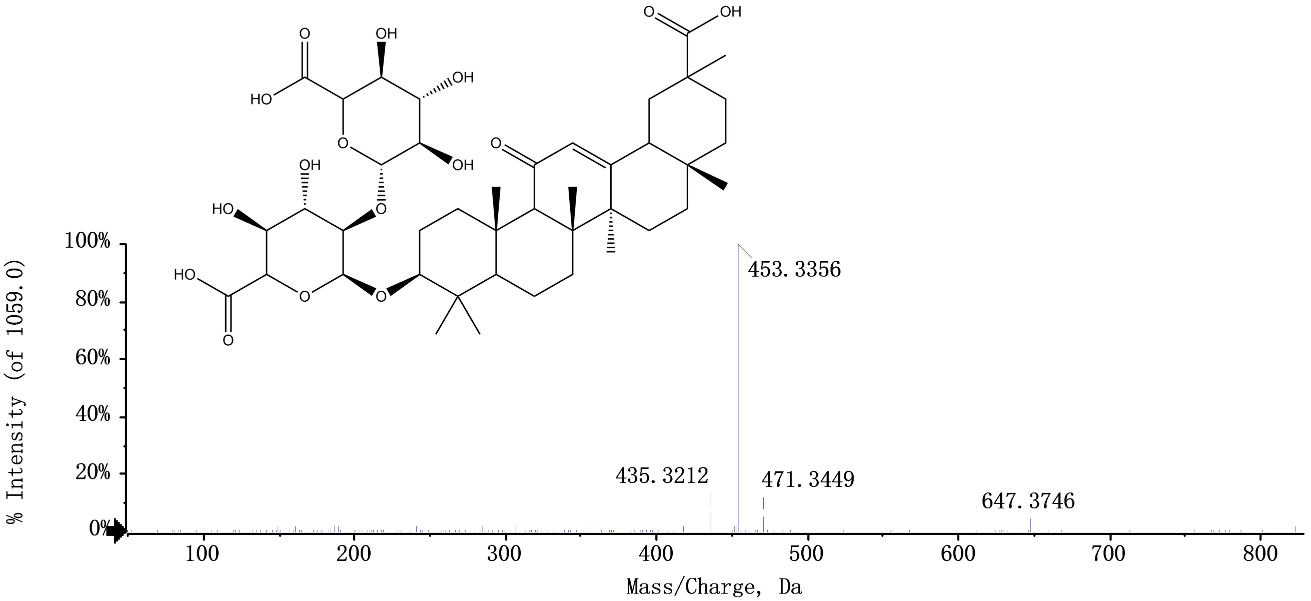

Figure S36A The positive MS/MS spectra of compound 36

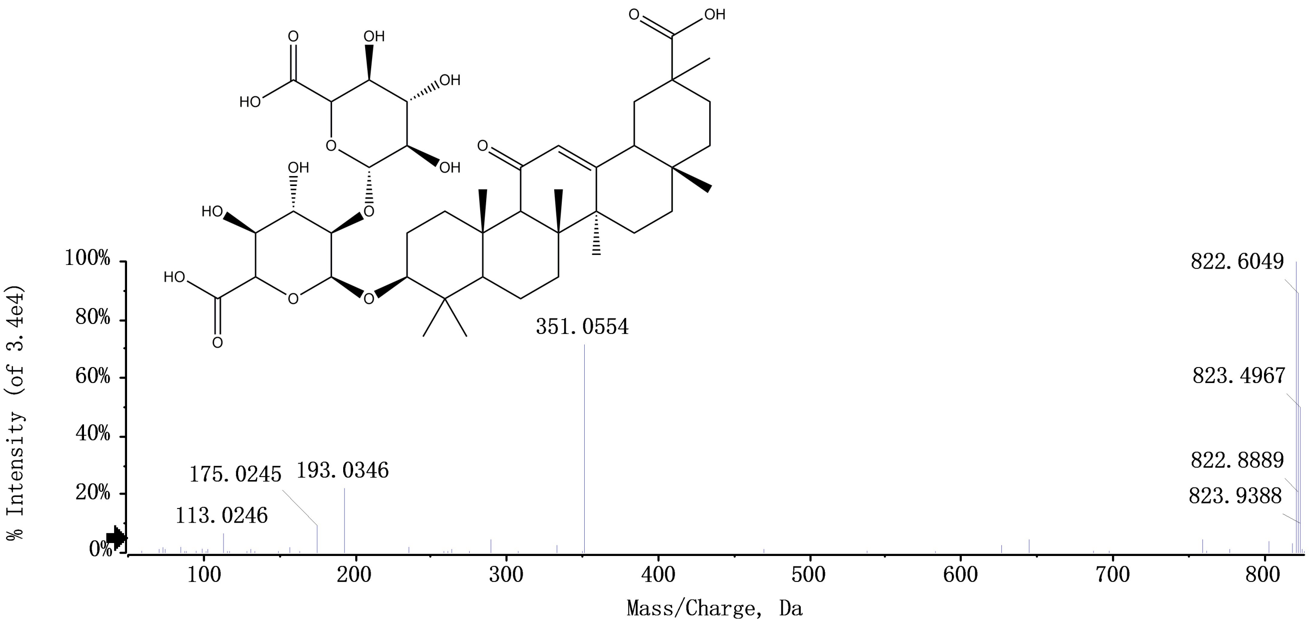

Figure S36B The negative MS/MS spectra of compound 36
